# Atypical local brain connectivity in pediatric autism spectrum disorder? A coordinate-based meta-analysis of regional homogeneity studies

**DOI:** 10.1007/s00406-022-01541-2

**Published:** 2023-01-04

**Authors:** Donato Liloia, Jordi Manuello, Tommaso Costa, Roberto Keller, Andrea Nani, Franco Cauda

**Affiliations:** 1 GCS-fMRI Research Group, Koelliker Hospital and Department of Psychology, University of Turin, Via Giuseppe Verdi 10, 10124 Turin, Italy; 2 Functional Neuroimaging and Complex Neural Systems (FOCUS) Laboratory, Department of Psychology, University of Turin, Turin, Italy; 3 Neuroscience Institute of Turin (NIT), Turin, Italy; 4 Adult Autism Center, DSM Local Health Unit, ASL TO, Turin, Italy

**Keywords:** fMRI, Resting state, Default mode network, Sensorimotor network, Seed-based d mapping, Neurosynth

## Abstract

Despite decades of massive neuroimaging research, the comprehensive characterization of short-range functional connectivity in autism spectrum disorder (ASD) remains a major challenge for scientific advances and clinical translation. From the theoretical point of view, it has been suggested a generalized local over-connectivity that would characterize ASD. This stance is known as the *general local over-connectivity* theory. However, there is little empirical evidence supporting such hypothesis, especially with regard to pediatric individuals with ASD (age $\le$ 18 years old). To explore this issue, we performed a coordinate-based meta-analysis of regional homogeneity studies to identify significant changes of local connectivity. Our analyses revealed local functional under-connectivity patterns in the bilateral posterior cingulate cortex and superior frontal gyrus (key components of the default mode network) and in the bilateral paracentral lobule (a part of the sensorimotor network). We also performed a functional association analysis of the identified areas, whose dysfunction is clinically consistent with the well-known deficits affecting individuals with ASD. Importantly, we did not find relevant clusters of local hyper-connectivity, which is contrary to the hypothesis that ASD may be characterized by generalized local over-connectivity. If confirmed, our result will provide a valuable insight into the understanding of the complex ASD pathophysiology.

**Supplementary Information:**

The online version contains supplementary material available at 10.1007/s00406-022-01541-2.

## Introduction

Autism spectrum disorder (ASD) is a cluster of neurobiological developmental conditions clinically evident from early childhood. ASD is characterized by a multifactorial etiology, with genetic, prenatal, and postnatal environmental factors playing a role [67, 99]. Though individuals with ASD have a heterogeneous phenotype with symptom severity ranging from mild to severe [92], the disorder is defined by the presence of persistent deficits in social interaction and communication, repetitive-restricted patterns of behavior or interests [24]. Medical comorbidities often co-occur in ASD, including other psychiatric conditions (e.g., attention-deficit/hyperactivity, anxiety, conduct, obsessive–compulsive, and depressive disorders), genetic disorders (e.g., dystrophinopathies, Fragile X and Down syndromes), or neurological conditions (e.g., epilepsy, learning disabilities, cerebral palsy) [1, 50]. The prevalence of ASD is estimated to be about 7.6 in 1000 individuals worldwide [9], with a female-to-male ratio around 1:3 [66]. ASD is currently diagnosed after the integration of information across multiple contexts and the administration of an array of standardized assessments, which include interview-based instruments and observational tools [68].

Although there is a substantial amount of biomedical literature on these conditions, the autistic pathophysiology is still under investigation and the identification of ASD through sound biomarkers remains an open challenge [67, 99]. In particular, the technology and protocols of functional magnetic resonance imaging (fMRI) have seen three decades of intense development, providing an unprecedented tool for *in-vivo* assessment of the neurophysiological basis of the disorder. Resting-state fMRI (rs-fMRI), an established corpus of methods capable of detecting spontaneous low-frequency regional temporal correlations in the blood oxygen level-dependent (BOLD) signal [13], has shown that brain functional connectivity in ASD is atypical throughout development [40, 113]. Previous research has focused extensively on aberrations of long-range connectivity and demonstrated patterns of hyper-connectivity in independent cohorts of pediatric subjects with ASD [82, 108, 117]. In contrast, adults with ASD are characterized by long-range patterns of hypo-connectivity, or no differences, compared to typically developing controls (TDCs) [75, 82, 112].

Despite evidence from multidisciplinary efforts suggesting focal cytoarchitectonic disorganizations in the autistic cerebral cortex [25], the presence of short-range functional connectivity abnormalities is less robustly established. Several authors have speculated that neuronal activity may be locally over-connected (i.e., the *general local over-connectivity* theory) [10, 20, 74, 97, 126]. However, there is little empirical evidence to support this hypothesis, especially due to the lack of effective and reliable computational fMRI metrics. Moreover, electroencephalography (EEG) and magnetoencephalography (MEG) studies investigating local electrophysiological connectivity in autistic groups have reported conflicting results [18, 85], in both children [26, 36] and adult [79] groups.

In recent years, independent studies have investigated atypical local short-range connectivity in ASD using regional homogeneity (ReHo), a whole-brain rs-fMRI technique that uses Kendall’s coefficient of concordance to test the coherence of time series of the BOLD signal amplitude in small clusters of neighboring voxels [47, 137]. Although ReHo was originally developed for cluster purification in fMRI data [137], its voxel-wise nature and high test–retest reliability [141] have provided important insights into the spatial extent of local connectivity in ASD, whose changes seem to be mainly localized in brain areas associated with visual [22, 44, 51, 73, 80], default mode [22, 23, 32, 59, 73, 80], salience [23, 87], and sensorimotor [22, 87] networks. Nonetheless, these findings are highly inconsistent. For example, several studies reported mixed patterns of local hypo- and hyper-connectivity [22, 23, 51, 73, 80, 87], while other studies found only lower or higher ReHo in ASD compared to TDCs [32, 44, 59]. Moreover, contradictory effects have been reported for areas such as the cerebellum [22, 87], middle frontal gyrus [87, 103], and insula [22, 32]. Of note, ReHo studies in ASD have focused almost exclusively on pediatric cohorts (age $\le$ 18 years). To our knowledge, only one study has examined ReHo changes in adults with ASD [22]. Unfortunately, this situation hinders a quantitative synthesis of local brain connectivity in ASD from a developmental life-long perspective [82, 113]. At the same time, however, it offers the opportunity to characterize short-range functional signatures in the critical time window in which ASD-related symptoms tend to emerge [96]. This may be important for the development of early neurobiology-based interventions, for example adopting meta-analytic identified areas for noninvasive brain stimulation [39, 65, 138].

In light of this context, this study aims to unravel for the first time, the most consistent and replicable patterns of ReHo changes in pediatric individuals with ASD and to test the hypothesis of *general local over-connectivity* in a data-driven manner. To this end, we used the Permutation-Subject Images version of Signed Differential Mapping (SDM-PSI) [4], one of the current coordinate-based meta-analytic methods that can provide a quantitative synthesis of neural changes across clinical groups [16, 56, 64, 107]. To further clarify the neurophysiological basis of ReHo aberrations, we examined the potential effects of clinical, sociodemographic, and methodological variables on published findings via voxel-wise meta-regression approach. Finally, we examined the large-scale network functional connectivity and psychological processes statistically related to the atypical ReHo clusters using the Neurosynth database [133], which allowed the interpretation of our findings from an observer-independent, unbiased perspective.

## Methods

The design of the study adheres to the current best-practice rules for neuroimaging coordinate-based meta-analyses (CBMAs) [72, 78, 109] and to the quality criteria of the PRISMA statement [90] (Table S1).

### Search strategy and data selection

A systematic literature search was performed in the PubMed database using the following combination of keywords: “*autism*” OR “*autism spectrum disorder*” OR “*ASD*” AND “*regional homogeneity*” OR “*ReHo*” OR “*local connectivity*”. Additionally, the reference lists of the included studies were manually checked and relevant reviews [40, 61] were inspected to identify articles that could have been missed during the dataset search. The final search was updated till January 2022, with no restrictions on publication year. For details see Table S2.

The identified articles were screened to verify their adherence with the following inclusion criteria: (1) to be an original article published in a peer-reviewed English-language journal; (2) to include one or more experiments investigating ReHo voxel-wise differences between subjects with ASD and TDCs at the whole-brain level; (3) to meet the diagnostic criteria of ASD based on the Autism Diagnostic Observation Schedule (ADOS) [69], Autism Diagnostic Interview-Revised (ADI-R) [70], Diagnostic and Statistical Manual of Mental Disorders (IV-R or 5 Edition) [5, 6], or the International Statistical Classification of Diseases and Related Health Problems 10th Revision [131]; (4) to include ASD and TDC subjects with an age at the scan session $\le$ 18 years; (5) to report significant results and coordinates (x–y-z) of clusters of ReHo changes using the Talairach (TAL) or Montreal Neurological Institute (MNI) stereotactic spaces.

Articles were excluded if: (1) they were case-report, conference abstracts or reviews; (2) they focused on animal models; (3) they did not report a between-group comparison (i.e., longitudinal studies without TDC groups) [72]; (4) they had sample sizes with fewer than 7 participants per group; (5) they performed ReHo analysis on a restricted region of the brain (i.e., ROI analysis) [78]; (6) they used no resting-state fMRI data (e.g., ReHo data derived from task-fMRI); (7) they explicitly indicated, for subjects with ASD, a co-occurring chronic systemic medical illness (i.e., other known neurologic, psychiatric, or genetic disorders). This choice is consistent with the need to characterize homogeneous clinical samples [109].

Particular attention was paid to avoiding spurious results due to overlap in the clinical population, both between and within articles. In the case of multiple experiments included in a single article, only those reporting on independent clinical groups were considered. In the case of multiple articles published using the same clinical group (or part of it), only the earliest published data set was considered.

### Data extraction

The articles were first extracted by one author (LD). The full-texts of the relevant articles were then independently evaluated by two authors (LD, MJ). Disagreements were resolved by consensus under the direction of the senior author (CF). Peak coordinates and related *T* values of abnormal ReHo clusters were extracted from all included experiments. When T-values were not provided, *Z*- or *P* values for significant clusters were converted to *T* values using the statistic converter utility of SDM (https://www.sdmproject.com/utilities/?show=Statistics).

### Statistical methods

#### Coordinate-based meta-analysis

Quantitative synthesis was performed using the signed differential mapping-permutation of subject images (SDM-PSI) software package (v.6.21). SDM-PSI is a recent CBMA method for neuroimaging that allows meta-analytic evaluation of independent results from voxel-wise neuroimaging studies; it benefits from the use of standard effect-size calculation, anisotropic Gaussian kernel approach and meta-analytic random-effect models [95]. The novelty of the method is the use of standard univariate voxel-wise tests [130], which, instead of identifying spatial convergence of the alteration across experiments, detect the presence or absence of the effect for each brain voxel. Full details on SDM-PSI can be found in Albajes-Eizagirre et al. [4].

Here we briefly summarize the procedure. The bounds (lower and upper) of the possible effect sizes for all voxels were evaluated with multiple imputations. Maps of ReHo changes for each study were generated using the anisotropic Gaussian kernel, which assigns higher effect sizes to voxels that appear to be more correlated with peak coordinates. We then applied maximum likelihood techniques to determine the most likely effect size and its standard error. This imputed data set obtained from each study was meta-analyzed with a random-effects model and, then, the obtained data sets were combined using Rubin’s rules. Finally, we performed family-wise error correction for multiple comparisons and thresholded our meta-analysis employing the threshold-free cluster enhancement statistics.

Preprocessing and mean analysis default thresholds were therefore adopted (i.e., Functional MRI modality; SDM gray matter mask; anisotropy = 1; isotropic FWHM = 20 mm; voxel size = 2 mm; number of imputations = 50). Results were corrected for multiple comparisons (family-wise error rate; 1,000 permutation runs). As recently recommended [4], threshold-free cluster enhancement (TFCE) [104] was used in statistical thresholding, setting a TFCE-based FWER *P* $\le$ 0.05 and a minimum cluster size of *k* = 10 voxels.

#### Heterogeneity and publication bias evaluation

The CBMA values of peak coordinates were extracted to direct heterogeneity statistics and publication bias analyses. We assessed heterogeneity between studies with the *I*^2^ statistic using a random-effects model, according to which an *I*^2^ < 50% is indicative of low heterogeneity [27]. We then performed funnel plots and Egger tests to estimate publication bias. An asymmetric plot and *p* < 0.05 were considered statistically significant.

#### Meta-regression analyses

The potential influences of clinical and methodological variables on ReHo findings were examined via meta-regression analysis. Heterogeneity between studies was explored for mean age at the scan session, gender distribution (i.e., percentage of female), cognitive functioning (i.e., FSIQ mean values), slice thickness and imaging smoothing level (i.e., FWHM), respectively. As suggested by the SDM team, a voxel-level threshold of P_uncorrected_ $\le$ 0.0005 was adopted to achieve an optimal balance between specificity and sensitivity [94].

#### Term associations and functional connectivity estimation

The probabilistic estimate of the association between voxels and significant psychological terms was derived from Neurosynth, a meta-analytic tool capable of retrieving results from more than 15,000 published fMRI studies using high-frequency keywords associated with fMRI voxel coordinates (https://github.com/neurosynth/neurosynth) [133]. This estimation is based on the probability that a certain psychological term is reported in association with the activation of a given voxel. The probabilistic estimate of Neurosynth can therefore be viewed as a quantitative indication of how activity in brain areas is functionally related to psychological processes. However, it is worth noting that Neurosynth does not distinguish between activations or deactivations of areas related to the term of interest. Even though Neurosynth reports more than 1,000 words, our focus was limited on cognitive and behavioral terms from the Cognitive Atlas [93], as recently suggested [37]. The resting-state functional connectivity was also estimated for each significant peak identified by our CBMA. We employed the 7-network atlas of Yeo et al. [135], who parceled the human cerebral cortex using rs-fMRI data from 1000 TDCs.

## Results

**Fig. 1:**
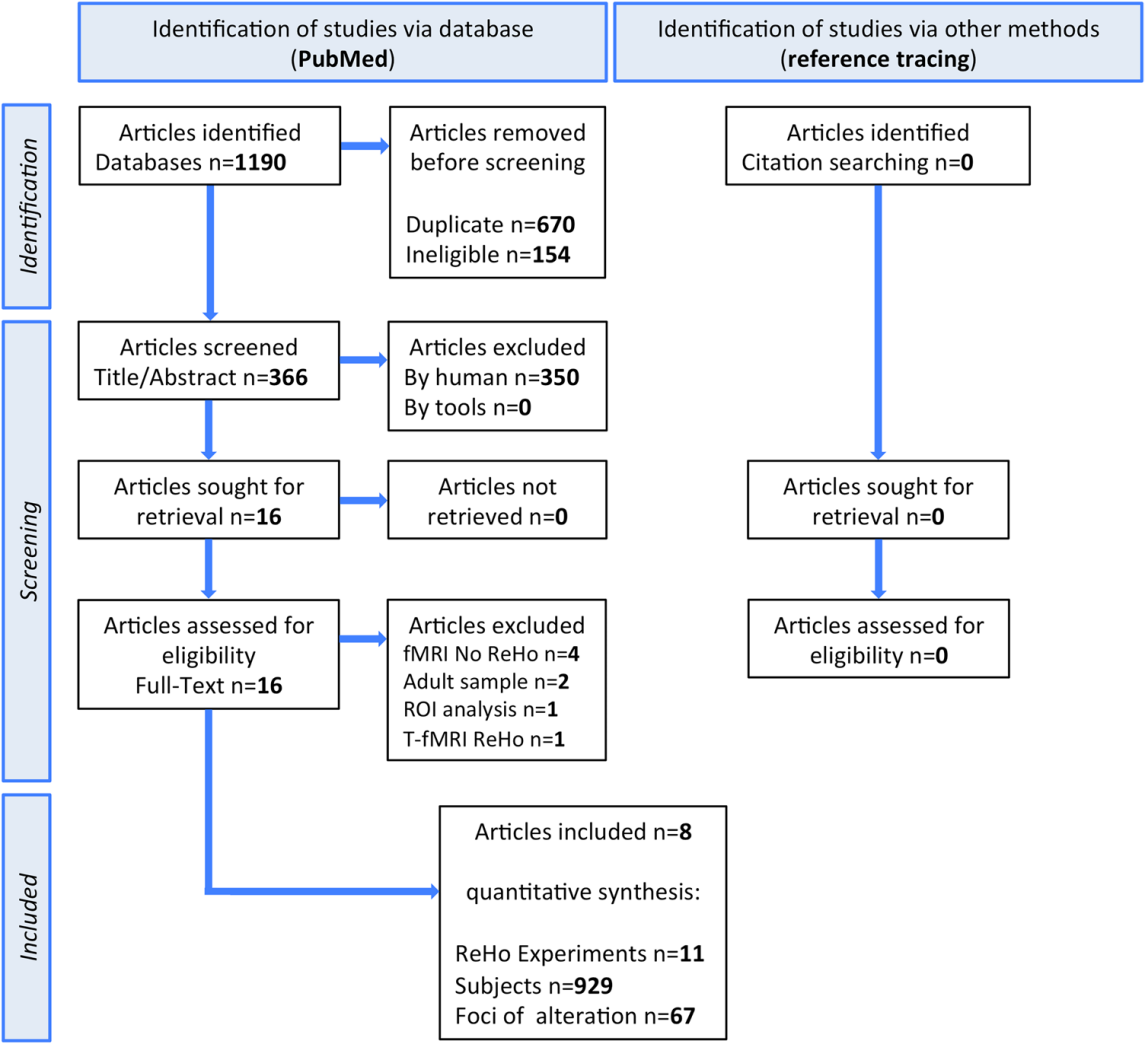
PRISMA flowchart for data selection in coordinate-based meta-analysis. *ReHo* regional homogeneity, *ROI* region of interest, *T-fMRI* task-based functional magnetic resonance imaging

The comprehensive literature search yielded 1190 articles. No additional articles were found in the reference lists of selected studies and relevant reviews. After title/abstract screening, 16 articles were evaluated at the full-text level, of which 8 were excluded based on our selective criteria. Four articles performed fMRI analyses that were not of interest [35, 42, 77, 121], 2 enrolled both pediatric and adult subjects in the experiment [23, 46], 1 performed an ROI analysis [48], 1 collected data while performing a visual task [103]. In total, 8 articles were included in the quantitative synthesis [22, 32, 44, 51, 59, 73, 80, 87], including 11 independent experimental datasets, 455 pediatric subjects with ASD (83 females and 372 males; mean age = 11.76 years), and 474 pediatric TDCs (110 females and 364 males; mean age = 11.94 years). No neurological, psychiatric, or genetic comorbidities were explicitly reported in the ASD groups of the selected ReHo experiments (details in Table S3). No article was excluded due to the presence of medical comorbidities in the ASD sample. The PRISMA flow diagram is shown in Fig. 1.

No significant differences were found between ASD and TDC groups with respect to age (mean confidence interval: − 0.179/0.090; *Z* = − 0.652, *P* = 0.514). For clinical-demographic and methodological details of included experiments, see also Table 1 and Table 2, respectively.

**Table 1:** Experiments included in the coordinate-based meta-analysis: demographic and clinical data

ASD						Controls					
First author	Sample	Age	Range	Diagnostic	FSIQ	Range	Sample	Age	Range	FSIQ	Range
(Experimental group)	(Female)	Mean (SD)		Tool	Mean (SD)		(Female)	Mean (SD)		Mean (SD)	
Dajani 2016 (children)	18 (1)	9.26 (1.28)	7.13–10.96	DSM-IV-TR; ADOS; ADI-R	112.44 (20.6)	84–148	18 (3)	9.32 (1.35)	7.19–10.86	112.72 (13.79)	80–138
Dajani 2016 (adolescent)	20 (4)	13.58 (1.86)	11.01–17.88	DSM-IV-TR; ADOS; ADI-R	104.55 (15.86)	78–132	20 (4)	14.28 (1.78)	11.03–17.7	104.95 (15.67)	80–134
Floris 2021	87 (44)	13.5 (2.8)	8.2–18	ADOS 2; ADI-R	101.5 (19.5)	70–145	109 (53)	13.7 (2.7)	8.2–17.9	111.5 (14.5)	79–143
Jao Keehn 2019	57 (10)	13.8 (2.6)	9.0–18	DSM-5; ADOS; ADI-R	104.4 (17.2)	66–141	51 (9)	13.2 (2.7)	8–17.6	106.4 (10.7)	79–126
Lan 2021	86 (0)	3.92 (0.95)	NA	DSM-5; CARS; ABC	53.44 (7.9)*	NA	54 (0)	4.09 (0.96)	NA	NA	NA
Li 2018	15 (0)	8.87 (3.11)	NA	DSM-5	50.47 (11.25)	NA	15 (0)	10.53 (2.61)	NA	127.27 (13.84)	NA
Maximo 2013	29 (4)	13.8 (2.4)	NA	ADOS; ADI-R	107.9 (19)	NA	29 (7)	13.5 (2.2)	NA	108 (8.9)	NA
Nair 2018 (SDSU)	26 (3)	13.93 (2.43)	9.2–17.7	DSM-IV-TR; ADOS; ADI-R	106.04 (18.47)	53–140	27 (5)	13.83 (2.26)	8.7–17.6	106.89 (17.19)	53–136
Nair 2018 (ABIDE-EO)	59 (6)	13.67 (2.6)	8–17.94	ADOS; ADI-R	102.43 (16.8)	64–137	82 (14)	13.7 (2.67)	8.39–17.9	102.32 (12.58)	76–127
Nair 2018 (ABIDE-EC)	30 (3)	13.33 (2.55)	7.15–17.17	ADOS; ADI-R	107.4 (14.87)	83–129	42 (6)	13.34 (2.4)	7.26–17.5	107.45 (13.21)	72–137
Paakki 2010	28 (8)	< 18	NA	ICD-10; ADOS; ADI-R	> 75	NA	27 (9)	< 18	NA	> 75	NA

*ASD* autism spectrum disorder, *FSIQ* full-scale intelligent quotient, *DSM* diagnostic and statistical manual of mental disorders, *ADOS* autism diagnostic observation schedule, *ADI* autism diagnostic interview, *CARS* childhood autism rating scale, *ABC* autistic behavior checklist, *ICD* international classification of disease, *NA* data not available.

* = developmental quotient

**Table 2:** Experiments included in the coordinate-based meta-analysis: methodological data

Study	Repetition	Slice	FWHM	Scanner	Threshold	GSR	Eyes	Cluster size	Stereotactic	ReHo changes	
(Experimental group)	Time	Thickness	mm	Tesla			Status	On ReHo	Space	TDC > ASD	TDC < ASD
Dajani 2016 (children)	2000/15	4	6	3	Corrected	NA	Open	27	MNI	3	3
Dajani 2016 (adolescent)	2000/15	4	6	3	Corrected	NA	Open	27	MNI	3	3
Floris 2021	2000/30	4	6	3	Corrected	NO	NA	27	MNI	2	0
Jao Keehn 2019	2000/30	4	6	3	Corrected	NO	Open	27	MNI	0	1
Lan 2021	2000/30	3.6	8	3	Corrected	NA	NA	27	MNI	4	1
Li 2018	2000/30	3	4	3	Corrected	NA	Closed	NA	MNI	0	2
Maximo 2013	2000/30	3.4	6	3	Corrected	NO	Open	27	MNI	7	4
Nair 2018 (SDSU)	2000/30	3.4	6	3	Corrected	NO	Open	27	MNI	5	3
Nair 2018 (ABIDE-EO)	NA	NA	6	3	Corrected	NO	Open	27	MNI	2	2
Nair 2018 (ABIDE-EC)	NA	NA	6	3	Corrected	NO	Closed	27	MNI	5	2
Paakki 2010	1800/40	4	4	1.5	Corrected	NO	Open	27	BRETT	6	9

*FWHM* full width at half maximum, *GSR* global signal regression, *MNI* Montreal neurological institute, *TAL* Talairach, *NA* data not available

Our dataset included three articles analyzing pediatric subjects from the Autism Brain Imaging Data Exchange database [23]. Therefore, only ReHo experiments with no overlap in the clinical population (both between and within articles) were analyzed. Further details can be found in Table S4.

**Fig. 2:**
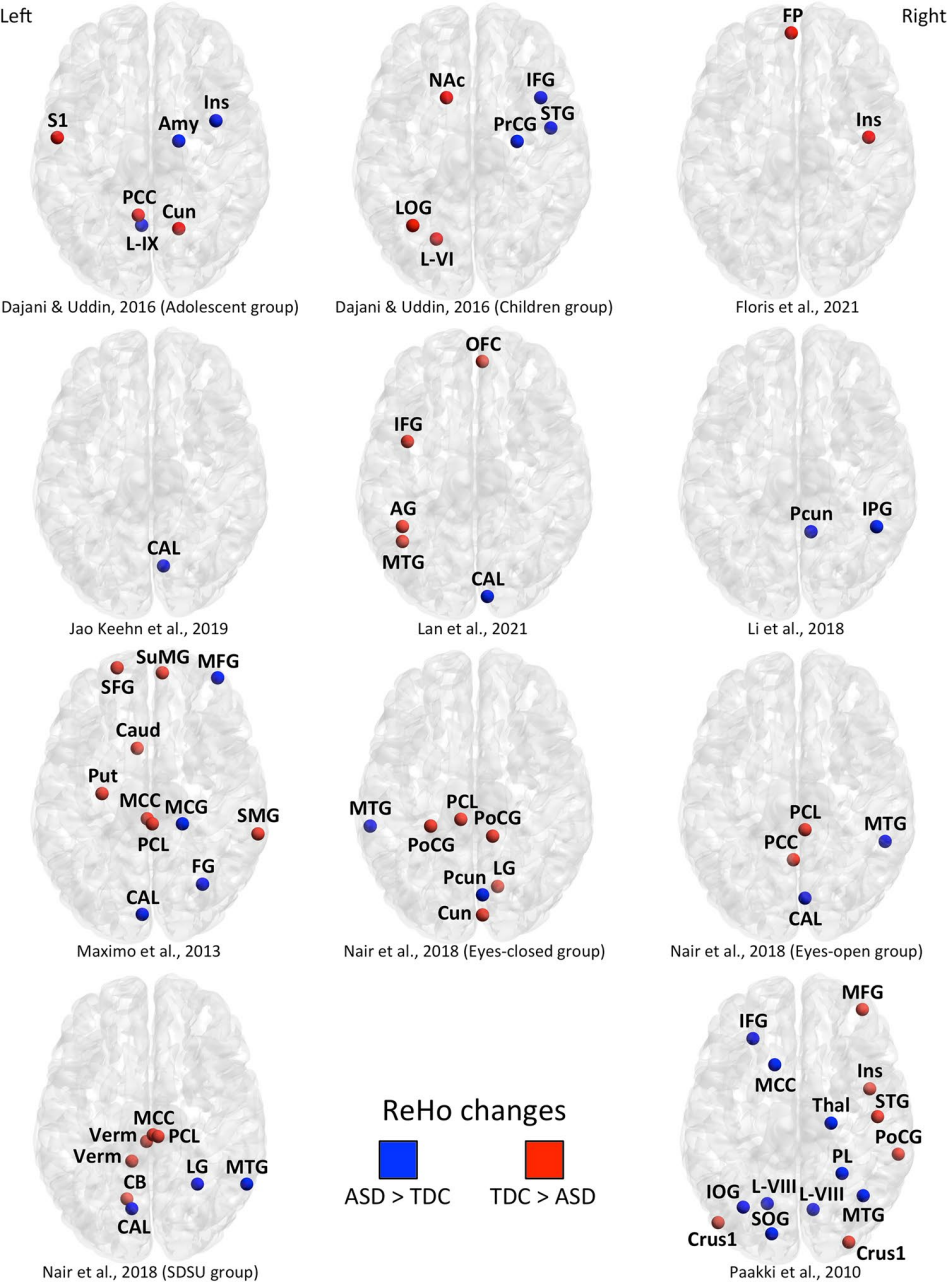
Anatomical distribution of stereotactic coordinates for each experiment included in the present coordinate-based meta-analysis. Nodes in blue reflect a significant regional homogeneity increase in pediatric subjects with autism spectrum disorder compared to typically developing controls. Nodes in red reflect a significant regional homogeneity decrease in pediatric subjects with autism spectrum disorder compared to typically developing controls. *ASD* autism spectrum disorder, *TDC* typically developing control, *AG* angular gyrus, *Amy* amygdala, *CAL* calcarine cortex, *Caud* caudate, *Crus1* cerebellar crus I, *Cun* cuneus, *FG* fusiform gyrus, *FP* frontal pole, *IFG* inferior frontal gyrus, *Ins* insula, *IOG* inferior occipital gyrus, *IPG* inferior parietal gyrus, *L-VI* cerebellar lobule VI, *L-VIII* cerebellar lobule VIII, *L-IX* cerebellar lobule IX, *LG* lingual gyrus, *LOG* lateral occipital gyrus, *MCC* middle cingulate cortex, *MCG* middle cingulate gyrus, *MFG* middle frontal gyrus, *MTG* middle temporal gyrus, *Nac* nucleus accumbens, *OFC* orbitofrontal cortex, *PCC* posterior cingulate cortex, *PCL* paracentral lobule, *Pcun* precuneus, *PL* parietal lobe, *PoCG* posterior central gyrus, *PrCG* precentral gyrus, *Put* putamen, *S1* primary somatosensory cortex, *SFG* superior frontal gyrus, *SMG* supramarginal gyrus, *SOG* superior occipital gyrus, *STG* superior temporal gyrus, *SuMG* superior medial gyrus, *Thal* thalamus, *Verm* verebellar vermis

To note, 67 localized peaks of ReHo changes were found to involve cortical, subcortical and cerebellar regions. Both higher and lower ReHo in the pediatric ASD groups compared with the TDC groups were observed (see also Fig. 2 for the spatial distribution of coordinates included in the current study).

### ReHo changes in pediatric ASD

**Fig. 3:**
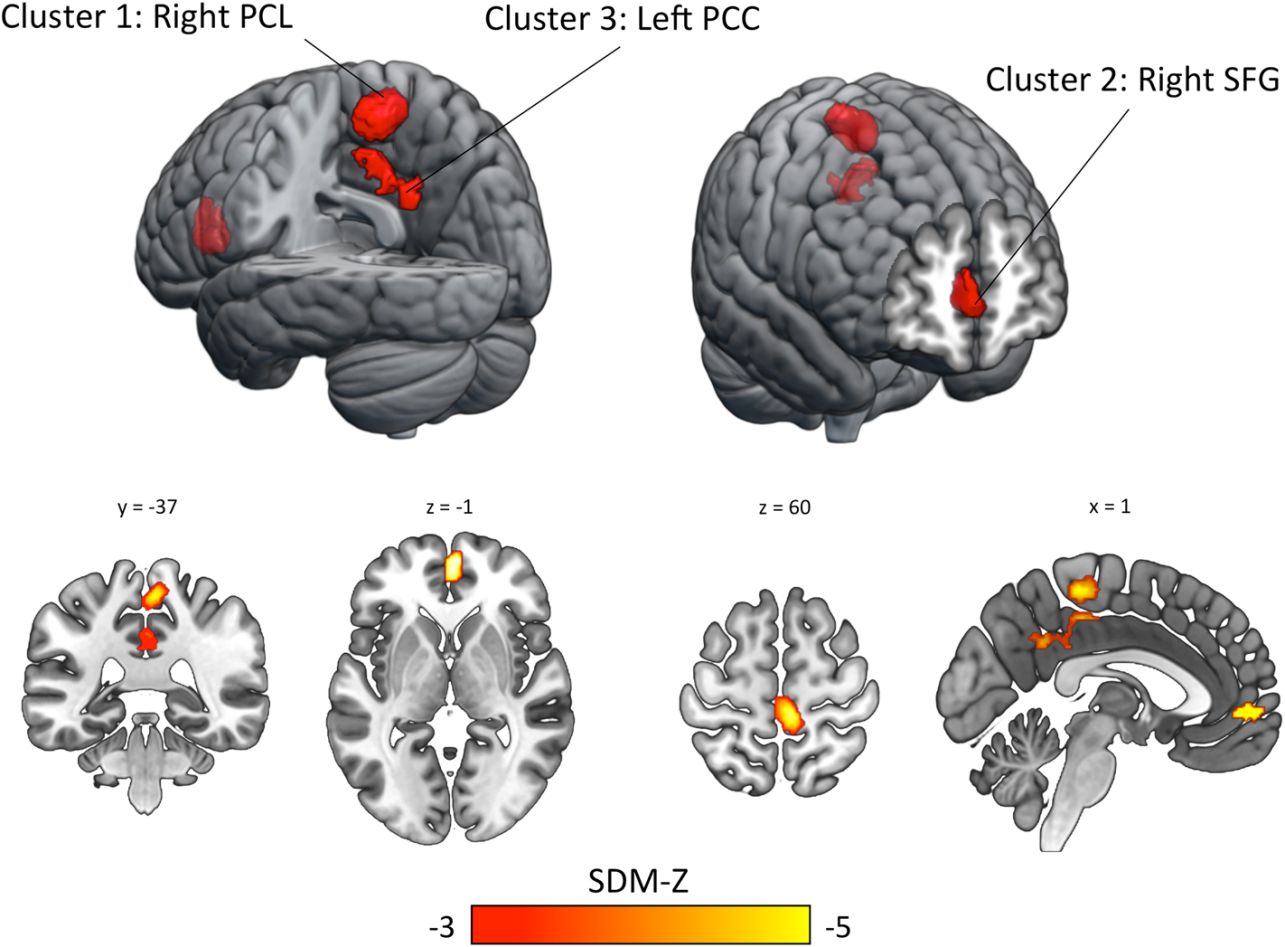
Brain clusters of regional homogeneity reduction in pediatric subjects with autism spectrum disorder compared to typically developing controls. Results are TFCE-based FWER corrected at 0.05. The PSI-SDM findings are visualized as hemispheric surfaces (3-D view) (upper panel) and coronal/axial/sagittal slices (2-D cortical and subcortical view) (lower panel). *PCL* paracentral lobule, *PCC* posterior cingulate cortex, *SFG* superior frontal gyrus

Compared with TDCs, pediatric subjects with ASD showed three clusters of ReHo decrease involving both hemispheres with an altered total volume of 6,160 mm^3^ (Table 3 and Fig. 3). Local peaks were found in the: (cluster 1) right paracentral lobule (PCL; Brodmann area—BA 4), which is part of the largest cluster along with the left paracentral lobule and right supplementary motor area (SMA); (cluster 2) right superior frontal gyrus (SFG; medial orbital part), which extends to the left SFG and bilateral anterior cingulate cortex (ACC; BA 10); (cluster 3) left posterior cingulate cortex (PCC; BA 23), which extends to the left precuneus and bilateral median cingulate cortex (MCC).

**Table 3:** Clusters of regional homogeneity reduction in pediatric subjects with autism spectrum disorder compared with typically developing controls

Region	MNI coordinate			SDM	*P*	Voxels	Cluster breakdown
	x	y	z	Z score	(Corrected)		(Voxels)
ASD < TDCs							
Right paracentral lobule (BA 4)	6	− 32	60	− 4.708	0.003	386	Right PCL BAs 4/5 (200) Left PCL BA 4 (141) Right SMA BA 4 (45)
Right superior frontal gyrus (BA 10)	2	56	− 2	− 5.082	0.004	205	Right SFG BA 10 (127) Left ACC BA 10 (36) Left SFG BA 10 (32) Right ACC BA 10 (10)
Left posterior cingulate cortex (BA 23)	− 2	− 50	32	− 3.726	0.032	179	Left MCC BA 23 (70) Left PCC BA 23 (41) Right MCC BA 23 (38) Left PCUN BA 23 (30)

For each cluster obtained, extrema Z-score, anatomic labels of the peaks of probability and its stereotactic coordinates were provided

*ASD* autism spectrum disorder, *TDCs* typically developing controls, *BA* Brodmann area, *MNI* Montreal Neurological Institute, *SDM* Seed-based d Mapping, *PCL* paracentral lobule, *SFG* superior frontal gyrus, *ACC* anterior cingulate cortex, *MCC* median cingulate cortex, *PCC* posterior cingulate cortex, *PCUN* precuneus

Note that a single cluster of ReHo increase encompassing the calcarine cortex (BA 17) bilaterally was found at *P* = 0.005 (Table S5; Fig. S1); however, it did not survive at the TFCE-based FWE correction.

### Analysis of heterogeneity and publication bias

Supplemental analyses revealed no significant heterogeneity of effect sizes in the current CBMA (*I*^2^ = 1.2 for peak 1; *I*^2^ = 1.0 peak 2; *I*^2^ = 5.7 peak 3). The results of Egger’s test and funnel plots revealed no obvious publication bias (*P* = 0.641 for peak 1; *P* = 0.755 peak 2; *P* = 0.474 peak 3).

### Effects of clinical and methodological variables

Several moderators were examined to understand between-study heterogeneity on published ReHo findings. No linear associations were found with FSIQ, age, gender distribution, smoothing, and slice thickness at P_uncorrected_ $\le$ 0.0005.

### Functional associations and connectivity

**Fig. 4:**
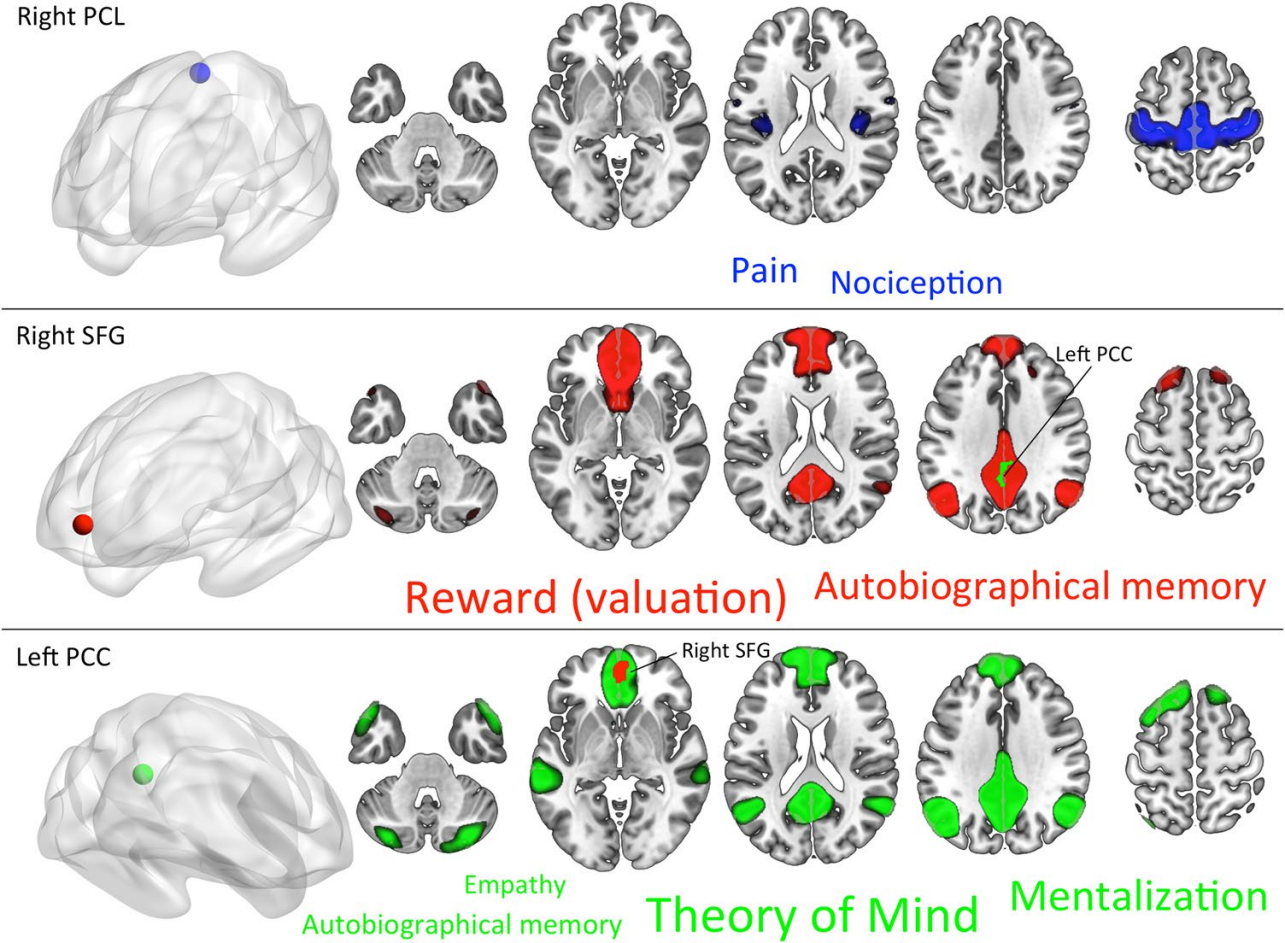
Psychological term associations and large-scale functional connectivity that are preferentially related to the peaks of regional homogeneity reduction in pediatric subjects with autism spectrum disorder compared to typically developing controls. Findings were generated using the Neurosynth database. Font size of the terms represents their associated Z-scores. *PCL* paracentral lobule, *SFG* superior frontal gyrus, *PCC* posterior cingulate cortex

According to the term association and connectivity analyses of the Neurosynth database, the right PCL is functionally associated with psychological processes of *pain* and *nociception* (see also Table S6 for the concept definitions provided by the Cognitive Atlas). According to the rs-fMRI atlas of Yeo et al. [135], its reliable co-activation is with areas of the sensorimotor network (Fig. 4). The right SFG is associated with psychological terms of *reward (evaluation)* and *autobiographical memory*. The right PCC is associated with the *Theory-of-Mind*, *mentalization*, *autobiographical memory*, and *empathy* terms (Table S6). The large-scale functional connectivity of both nodes suggests a strong involvement of the default mode network [135] (Fig. 4).

## Discussion

This study provides a unique quantitative synthesis of resting-state ReHo changes in pediatric individuals with ASD. Taking advantage of the current state-of-the-art methods in the field of CBMA, we revealed consistent patterns of local functional under-connectivity across included experiments, predominantly within the default mode and sensorimotor networks. These findings were highly reliable according to the heterogeneity, publication bias, and meta-regression analyses. Moreover, data-driven characterization of the identified areas revealed both sensory and high-level psychological associations that have been widely documented as deficient in the disorder. Altogether, these results are an important first step in resolving discrepancies in the ReHo literature of ASD and, at the same time, emphasize the necessity of reconsidering the theoretical hypothesis of the *generalized local over-connectivity* in pediatric autism.

An important finding of this investigation is that consistent patterns of local hypo-connectivity accumulate in the core components of the functionally defined DMN, namely the SFG (medial orbital part) and PCC [115]. This result accords well with the growing evidence for a significant contribution of DMN dysmaturation to the pathophysiology of ASD [38, 55, 61, 63, 88]. Both regions are implicated in the disorder, including aberrations in gray matter volume/concentration [63, 116], cortical thickness [12, 118], white matter connectivity [83, 122], intrinsic functional connectivity [71, 127], and task-related activation [49, 128]. It is worth noting that the long-range hypo-connectivity between the PCC and SFG is one of the most widely replicated findings in the fMRI literature on autism [14, 55] and is thought to underlie reduced integration of self- and other-referential processing in children with ASD [14]. These same sites are classified as *hub* nodes of the human connectome due to their high degree of anatomo-functional connections and pivotal role in maintaining global brain communication [81, 106, 140]. They also have been repeatedly found abnormal in resting-state activity in a wide range of clinical conditions, including depression [43], attention-deficit hyperactivity disorder [45], mild cognitive impairment [53], Parkinson’s disease [91], and schizophrenia [132]. Given these observations, our results are consistent with the cross-disorder dysconnectivity model proposed by Van den Heuvel and Sporns [119], which suggests a possible shared landscape of alteration in the functional connectome across multiple diseases, particularly affecting brain areas characterized by high topological centrality [21] and metabolic activity [110].

The lower regional homogeneity of the PCC is an interesting finding for future research on the role of this brain area in the disorder. This finding extends previous postmortem evidence of altered distribution of neurons within the PCC in pediatric individuals with ASD [30, 86] and is well consistent with the results of EEG research highlighting the PCC as one of the central nodes of disconnectivity in the autistic brain [125]. In this context, it is worth noting that this area is considered the main hub of the DMN due to its greatest functional centrality, which begins to emerge in 2-week-olds typically developing individuals [34]. Despite the broad consensus on its cognitive importance, the exact functional profile of the PCC has not been fully elucidated. The PCC is associated with self-awareness, detection of behaviorally relevant environmental changes, internally directed thought, and regulation of internal and external focuses of attention [58]. This picture is strongly consistent with our functional association analysis, in which high-level psychological terms were associated with the right PCC as well as with the typical clinical impairment of ASD. From a clinical perspective, dysfunction of the PCC has been associated with deficits in the *Theory-of-Mind* (*ToM*; i.e., the ability to infer people’s emotional states, thoughts or beliefs), *mentalization* (i.e., the ability to understand the mental state of others or oneself), and *empathy* (i.e., the ability to be sensitive to people’s feelings). The scientific literature showing that individuals with ASD have significant deficits in these interrelated functions is extensive and robust, e.g. [8, 17, 105]. From a neuroimaging perspective, a number of studies suggest that individuals with ASD exhibit abnormal activation and connectivity in certain DMN regions (i.e., PCC, precuneus, angular gyrus, and medial prefrontal cortex), thought to play a role in tasks related to ToM, empathy, and mentalizing [29, 31, 41, 49, 101, 128].

Another area that exhibited lower regional homogeneity was the SFG. Considering the conflicting results of the EEG and MRI literature on functional connectivity in ASD [85, 113, 123], the detection of short-range under-connectivity in this site is particularly noteworthy. Interestingly, in the mouse model with 16p11.2 deletion, one of the most common chromosomal copy number variations in ASD, the SFG was associated with disrupted functional connectivity [11]. In the same line of research, one study found that loss of the scaffolding protein SHANK3, which is commonly associated with ASD, can lead to impaired functional connectivity and abnormal neuroanatomical structures in prefrontal areas [89]. Although it is tempting to speculate that these genetic impairments might affect prefrontal functional connectivity in individuals with ASD, further multimodal research is needed. Moreover, recent research has revealed that thousands of genomic risk variants profoundly impact functional brain connectivity in a set of psychiatric conditions, including ASD [76].

The SFG disconnectivity may be predictive of deficits in social communication and interpersonal interaction, which, once again, is a distinctive feature of ASD [114]. Moreover, functional association analysis links this area and other frontal components especially to the processes of *autobiographical memory* and *reward*. There is evidence that the experience of recollecting personal events is reduced in ASD [19]. In particular, the deficit regards the ability of retrieving memories as well as of reconstructing autobiographical records. This, in turn, may lead to difficulties in simulating future scenarios, as this ability requires a system that can rely on past experiences, so as to identify and distinguish relevant elements that can be used to predict future outcomes [100]. The prefrontal cortex also plays an important role in modulating the reward process [15]; its dysfunction contributes to disrupting corticostriatal pathways, which may lead to the apparent difficulties in experiencing social reward that affect people with ASD [60]. Finally, growing evidence points out that changes of ReHo most likely induce alterations of remote functional connectivity [47]. Therefore, our findings of lower regional homogeneity of PCC and SFG further support the increasing body of experimental inquiries indicating aberrant functional network synchronization in ASD when frontal or parietal components are affected, as dysregulation of these areas may lead to reduced long-range coupling between prefrontal and parietal associative regions.

A consistent pattern of local hypo-connectivity was also revealed in a mainly right-sided cluster that included the PCL. The emerging picture of this U-shaped convolution, located between the precentral and postcentral gyri, provides an important framework for interpreting the clinical significance of our results. Indeed, this node of the sensorimotor network exhibits altered functional connectivity in ASD, particularly in the early stages of the disorder [33, 39, 57, 73, 80, 136]. Moreover, this area has been proposed as a site for noninvasive brain stimulation to alleviate sensory symptoms in pediatric individuals with ASD [39]. Previous studies have shown that this highly interconnected region is involved in pain-evoked activity, aversive emotional processing, and painful sensations [98, 102, 139]. Consistent with these reports, Neurosynth analysis highlights a statistical association of the right PCL to the psychological processes of *pain* and *nociception*. This finding, although indirect, is important because the role of aberrant processing and sensitivity in ASD is highly understudied, especially in children and adolescents [129, 134]. Still, considering that both sensory symptoms occur in approximately 69% of children with ASD [7], and that pain-related levels may be considered as a predictor of poor health outcome in adolescents with autism [111], there is a need for future fMRI research that directly addresses the neural pain signature in this disorder, for example, by adopting our coordinates as regions-of-interest.

Our findings are only partially consistent with those of previous CBMAs on rs-fMRI of ASD [54, 124]. In particular, we revealed hypo-connectivity at the level of the PCL and PCC clusters in line with Lau et al. [54], even though their result showed a more extensive volume of alteration and that only 3 out of the 11 experiments included here were previously meta-analyzed by the authors. In contrast to Wang et al. [124], we did not replicate the findings of hypo-connectivity at the level of the right middle temporal gyrus and bilateral cerebellar crus I, and hyper-connectivity in the left precentral gyrus, right inferior frontal gyrus and bilateral cerebellar lobule IX. In addition, we detected a cluster of hypo-connectivity in the SFG that was not found previously.

These discrepancies could be explained by several factors. First, previous investigations have adopted a multimodal resting-state perspective, that is, they have synthetized findings from ReHo, arterial spin labeling (ASL), independent component analysis (ICA), and amplitude of low-frequency fluctuations (ALFF) techniques. Of note, Wang et al. [124] also used coordinates from positron emission tomography (PET) and single-photon emission computed tomography (SPECT) experiments. Second, Lau et al. [54] and Wang et al. [124] used the activation likelihood estimation and effect-size version of SDM, respectively. These CBMA methods test for spatial convergence of alteration across coordinates. In contrast, our CBMA method performs standard univariate voxel-wise tests. From a methodological perspective, this means that SDM-PSI is able to overcome certain spatial drawbacks that can lead to either conservative or liberal results and reduce the statistical power of CBMA [2, 4]. Third, early CBMAs have summarized findings from a pediatric, adult, and mixed-age groups altogether. Although this is a valuable choice that provides reliable results in terms of generalization to the clinical condition of interest, it limits the accurate characterization of the autistic brain phenotype, which is known to differ across neurodevelopmental stages [14, 22, 52, 62, 82, 84, 113].

We found no significant clusters of local hyper-connectivity. This is an unexpected result considering that 10 of the 11 included experiments reported at least one coordinate of ReHo increase. Probably, a moot point is a calcarine cortex that, although locally hyperconnected in a number of studies [44, 51, 73, 80], did not survive our rigorous statistical thresholding procedure. One possible reason for this result is that the exact loci of local maximum differed considerably across samples. This and the fact that our design used x–y-z coordinates instead of three-dimensional parametric maps could explain why no effect was detected in this study. Another aspect could be related to the status of the participants’ eyes during MRI acquisition. As Nair et al. [80] have elegantly shown, ReHo measurements may be susceptible to eye openness/closure due to differential effects on local activity synchronization, especially in the visual-related regions. Unfortunately, the limited number of experiments, as well as the lack of specification of this variable in some studies, has hampered the ability to perform subanalyses on this topic. Therefore, further research is needed to better characterize this confounding factor.

Finally, it is worth noting that our findings do not support the currently prevailing theory of *general local over-connectivity* in ASD. Starting from the review of Courchesne and Pierce [20], several authors have proposed that the autistic brain, and in particular the frontal cortex, is characterized by excessive connectivity and disorganized heightened excitability. However, upon further review, we note that this hypothesis has survived over time via cross-citations [74, 97, 120, 126] and was based primarily on findings from anatomical and post-mortem microscopic studies due to the limited availability of rs-fMRI research data at the time of its first conception. Therefore, at least in pediatric individuals, ReHo research indicates that the notion of local over-connectivity needs to be reconsidered based on current data.

### Limitations and future challenges

The current findings need to be contextualized with respect to some limitations. CBMA methods, by definition, have limited accuracy because they employ stereotactic coordinates instead of original statistical parametric maps of alteration. On the other hand, we need to observe that the high standardization of this method may limit the probability of spatial errors [28, 94]. Also, current findings are based on 11 experiments due to the limited availability of published research data. In performing the SDM-PSI analysis, we cannot rule out the possibility that considering a small set of experiments may bias the effect sizes slightly towards zero, even though a simulation using an algorithm with the maximum likelihood/multiple imputations has shown that this type of bias is almost negligible [3]. Meta-regression analyses revealed no apparent heterogeneity in demographic-clinical-methodological variables between experiments. These results are based only on a limited number of data and, therefore, should be taken with caution and should be confirmed with further studies. As highlighted throughout the text, the converging MRI-based literature suggests that functional brain connectivity in ASD should be characterized from a developmental perspective [22, 82, 113]. This study contributes in part to this view due to its cross-sectional nature and circumscribed focus on pediatric cohorts. Future ReHo research in adults with autism, as well as longitudinal studies examining the same individuals across the lifespan, is urgently needed to understand the precise developmental trajectory of local connectivity in ASD. Finally, one of the long-term goals of the fMRI connectivity approaches in ASD is to provide valuable insights for clinical practice. Therefore, future investigations could enroll autistic subjects with other common medical comorbidities to test in detail the impact of these co-occurrences on the brain landscape of people with ASD.

## Conclusions

Our analysis of resting-state ReHo changes in pediatric individuals with ASD provides valuable insight into an area that remains poorly explored. Somewhat unexpectedly, no significant local hyper-connectivity was found, despite the existing hypothesis of *generalized local over-connectivity*. On the contrary, patterns of local functional hypo-connectivity were observed mainly in the bilateral PCC and SFG, as well as in the bilateral PCL. Functional characterization of these regions revealed associations with sensory and socio-executive domains known to be affected by ASD. Thus, our results provide an insightful step toward a better understanding of the complex pathophysiology of this multifaceted spectrum.

### Supplementary Information

Below is the link to the electronic supplementary material.

Supplementary file1 (DOCX 668 KB)

## Data Availability

Foci of alteration and PSI-SDM maps are available upon reasonable request.

## References

[1] Al-Beltagi M (2021). Autism medical comorbidities. World J Clin Pediatr.

[2] Albajes-Eizagirre A, Radua J (2018). What do results from coordinate-based meta-analyses tell us?. Neuroimage.

[3] Albajes-Eizagirre A, Solanes A, Radua J (2019). Meta-analysis of non-statistically significant unreported effects. Stat Methods Med Res.

[4] Albajes-Eizagirre A, Solanes A, Vieta E, Radua J (2019). Voxel-based meta-analysis via permutation of subject images (psi): theory and implementation for sdm. Neuroimage.

[5] American Psychiatric Association (2013). Diagnostic and statistical manual of mental disorders, fifth edition (dsm-5).

[6] American Psychiatric Association (2000). Diagnostic and statistical manual of mental disorders, fourth edition, text revision (dsm-iv-tr®).

[7] Baranek GT, David FJ, Poe MD, Stone WL, Watson LR (2006). Sensory experiences questionnaire: discriminating sensory features in young children with autism, developmental delays, and typical development. J Child Psychol Psychiatry.

[8] Baron-Cohen S, Leslie AM, Frith U (1985). Does the autistic child have a “theory of mind” ?. Cognition.

[9] Baxter AJ, Brugha TS, Erskine HE, Scheurer RW, Vos T, Scott JG (2014). The epidemiology and global burden of autism spectrum disorders. Psychol Med.

[10] Belmonte MK, Allen G, Beckel-Mitchener A, Boulanger LM, Carper RA, Webb SJ (2004). Autism and abnormal development of brain connectivity. J Neurosci.

[11] Bertero A, Liska A, Pagani M, Parolisi R, Masferrer ME, Gritti M, Pedrazzoli M, Galbusera A, Sarica A, Cerasa A, Buffelli M, Tonini R, Buffo A, Gross C, Pasqualetti M, Gozzi A (2018). Autism-associated 16p11.2 microdeletion impairs prefrontal functional connectivity in mouse and human. Brain.

[12] Bieneck V, Bletsch A, Mann C, Schäfer T, Seelemeyer H, Herøy N, Zimmermann J, Pretzsch CM, Hattingen E, Ecker C (2021). Longitudinal changes in cortical thickness in adolescents with autism spectrum disorder and their association with restricted and repetitive behaviors. Genes.

[13] Buckner RL, Krienen FM, Yeo BT (2013). Opportunities and limitations of intrinsic functional connectivity mri. Nat Neurosci.

[14] Burrows CA, Laird AR, Uddin LQ (2016). Functional connectivity of brain regions for self- and other-evaluation in children, adolescents and adults with autism. Dev Sci.

[15] Chau BKH, Jarvis H, Law CK, Chong TT (2018). Dopamine and reward: a view from the prefrontal cortex. Behav Pharmacol.

[16] Chavanne AV, Robinson OJ (2021). The overlapping neurobiology of induced and pathological anxiety: a meta-analysis of functional neural activation. Am J Psychiatry.

[17] Chung YS, Barch D, Strube M (2014). A meta-analysis of mentalizing impairments in adults with schizophrenia and autism spectrum disorder. Schizophr Bull.

[18] Coben R, Mohammad-Rezazadeh I, Cannon R (2014). Using quantitative and analytic eeg methods in the understanding of connectivity in autism spectrum disorders: a theory of mixed over- and under-connectivity. Front Hum Neurosci.

[19] Cooper RA, Simons JS (2019). Exploring the neurocognitive basis of episodic recollection in autism. Psychon Bull Rev.

[20] Courchesne E, Pierce K (2005). Why the frontal cortex in autism might be talking only to itself: Local over-connectivity but long-distance disconnection. Curr Opin Neurobiol.

[21] Crossley NA, Mechelli A, Scott J, Carletti F, Fox PT, McGuire P, Bullmore ET (2014). The hubs of the human connectome are generally implicated in the anatomy of brain disorders. Brain.

[22] Dajani DR, Uddin LQ (2016). Local brain connectivity across development in autism spectrum disorder: A cross-sectional investigation. Autism Res.

[23] Di Martino A, Yan CG, Li Q, Denio E, Castellanos FX, Alaerts K, Anderson JS, Assaf M, Bookheimer SY, Dapretto M, Deen B, Delmonte S, Dinstein I, Ertl-Wagner B, Fair DA, Gallagher L, Kennedy DP, Keown CL, Keysers C, Lainhart JE, Lord C, Luna B, Menon V, Minshew NJ, Monk CS, Mueller S, Müller RA, Nebel MB, Nigg JT, O'Hearn K, Pelphrey KA, Peltier SJ, Rudie JD, Sunaert S, Thioux M, Tyszka JM, Uddin LQ, Verhoeven JS, Wenderoth N, Wiggins JL, Mostofsky SH, Milham MP (2014). The autism brain imaging data exchange: Towards a large-scale evaluation of the intrinsic brain architecture in autism. Mol Psychiatry.

[24] Doernberg E, Hollander E (2016). Neurodevelopmental disorders (asd and adhd): Dsm-5, icd-10, and icd-11. CNS Spectr.

[25] Donovan AP, Basson MA (2017). The neuroanatomy of autism - a developmental perspective. J Anat.

[26] Duffy FH, Als H (2012). A stable pattern of eeg spectral coherence distinguishes children with autism from neuro-typical controls - a large case control study. BMC Med.

[27] Egger M, Davey Smith G, Schneider M, Minder C (1997). Bias in meta-analysis detected by a simple, graphical test. BMJ (Clinical research ed).

[28] Eickhoff SB, Laird AR, Grefkes C, Wang LE, Zilles K, Fox PT (2009). Coordinate-based activation likelihood estimation meta-analysis of neuroimaging data: a random-effects approach based on empirical estimates of spatial uncertainty. Hum Brain Mapp.

[29] Fan Y-T, Chen C, Chen S-C, Decety J, Cheng Y (2013). Empathic arousal and social understanding in individuals with autism: evidence from fmri and erp measurements. Soc Cognit Affect Neurosci.

[30] Fetit R, Hillary RF, Price DJ, Lawrie SM (2021). The neuropathology of autism: a systematic review of post-mortem studies of autism and related disorders. Neurosci Biobehav Rev.

[31] Fishman I, Keown CL, Lincoln AJ, Pineda JA, Müller R-A (2014). Atypical cross talk between mentalizing and mirror neuron networks in autism spectrum disorder. JAMA Psychiat.

[32] Floris DL, Filho JOA, Lai MC, Giavasis S, Oldehinkel M, Mennes M, Charman T, Tillmann J, Dumas G, Ecker C, Dell'Acqua F, Banaschewski T, Moessnang C, Baron-Cohen S, Durston S, Loth E, Murphy DGM, Buitelaar JK, Beckmann CF, Milham MP, Di Martino A (2021). Towards robust and replicable sex differences in the intrinsic brain function of autism. Mole Autism.

[33] Fu Z, Tu Y, Di X, Du Y, Sui J, Biswal BB, Zhang Z, de Lacy N, Calhoun VD (2019). Transient increased thalamic-sensory connectivity and decreased whole-brain dynamism in autism. Neuroimage.

[34] Gao W, Zhu H, Giovanello KS, Smith JK, Shen D, Gilmore JH, Lin W (2009). Evidence on the emergence of the brain's default network from 2-week-old to 2-year-old healthy pediatric subjects. Proc Natl Acad Sci.

[35] Guo X, Chen H, Long Z, Duan X, Zhang Y, Chen H (2017). Atypical developmental trajectory of local spontaneous brain activity in autism spectrum disorder. Sci Rep.

[36] Han YMY, Chan AS, Sze SL, Cheung M-C, Wong C-k, Lam JMK, Poon PMK (2013). Altered immune function associated with disordered neural connectivity and executive dysfunctions: a neurophysiological study on children with autism spectrum disorders. Res Autism Spectr Disord.

[37] Hansen JY, Markello RD, Vogel JW, Seidlitz J, Bzdok D, Misic B (2021). Mapping gene transcription and neurocognition across human neocortex. Nat Hum Behav.

[38] Harikumar A, Evans DW, Dougherty CC, Carpenter KLH, Michael AM (2021). A review of the default mode network in autism spectrum disorders and attention deficit hyperactivity disorder. Brain Connect.

[39] Huang Y, Zhang B, Cao J, Yu S, Wilson G, Park J, Kong J (2020). Potential locations for noninvasive brain stimulation in treating autism spectrum disorders-a functional connectivity study. Front Psych.

[40] Hull JV, Dokovna LB, Jacokes ZJ, Torgerson CM, Irimia A, Van Horn JD (2017). Resting-state functional connectivity in autism spectrum disorders: a review. Front Psychiatry.

[41] Hyatt CJ, Calhoun VD, Pittman B, Corbera S, Bell MD, Rabany L, Pelphrey K, Pearlson GD, Assaf M (2020). Default mode network modulation by mentalizing in young adults with autism spectrum disorder or schizophrenia. Neuroimage.

[42] Itahashi T, Yamada T, Watanabe H, Nakamura M, Ohta H, Kanai C, Iwanami A, Kato N, Hashimoto R (2015). Alterations of local spontaneous brain activity and connectivity in adults with high-functioning autism spectrum disorder. Mol Autism.

[43] Iwabuchi SJ, Krishnadas R, Li C, Auer DP, Radua J, Palaniyappan L (2015). Localized connectivity in depression: a meta-analysis of resting state functional imaging studies. Neurosci Biobehav Rev.

[44] Jao Keehn RJ, Nair S, Pueschel EB, Linke AC, Fishman I, Müller RA (2019). Atypical local and distal patterns of occipito-frontal functional connectivity are related to symptom severity in autism. Cereb Cortex.

[45] Jiang K, Yi Y, Li L, Li H, Shen H, Zhao F, Xu Y, Zheng A (2019). Functional network connectivity changes in children with attention-deficit hyperactivity disorder: a resting-state fmri study. Int J Dev Neurosci.

[46] Jiang L, Hou X-H, Yang N, Yang Z, Zuo X-N (2015). Examination of local functional homogeneity in autism. Biomed Res Int.

[47] Jiang L, Zuo XN (2016). Regional homogeneity: a multimodal, multiscale neuroimaging marker of the human connectome. The Neurosci.

[48] Jung M, Mody M, Saito DN, Tomoda A, Okazawa H, Wada Y, Kosaka H (2015). Sex differences in the default mode network with regard to autism spectrum traits: a resting state fmri study. PLoS ONE.

[49] Kana RK, Maximo JO, Williams DL, Keller TA, Schipul SE, Cherkassky VL, Minshew NJ, Just MA (2015). Aberrant functioning of the theory-of-mind network in children and adolescents with autism. Mol Autism.

[50] Lai M-C, Kassee C, Besney R, Bonato S, Hull L, Mandy W, Szatmari P, Ameis SH (2019). Prevalence of co-occurring mental health diagnoses in the autism population: a systematic review and meta-analysis. Lancet Psychiatry.

[51] Lan Z, Xu S, Wu Y, Xia L, Hua K, Li M, Liu M, Yin Y, Li C, Huang S, Feng Y, Jiang G, Wang T (2021). Alterations of regional homogeneity in preschool boys with autism spectrum disorders. Front Neurosci.

[52] Lange N, Travers BG, Bigler ED, Prigge MB, Froehlich AL, Nielsen JA, Cariello AN, Zielinski BA, Anderson JS, Fletcher PT, Alexander AA, Lainhart JE (2015). Longitudinal volumetric brain changes in autism spectrum disorder ages 6–35 years. Autism Res.

[53] Lau WK, Leung MK, Lee TM, Law AC (2016). Resting-state abnormalities in amnestic mild cognitive impairment: a meta-analysis. Transl Psychiatry.

[54] Lau WKW, Leung MK, Lau BWM (2019). Resting-state abnormalities in autism spectrum disorders: a meta-analysis. Sci Rep.

[55] Lau WKW, Leung MK, Zhang R (2020). Hypofunctional connectivity between the posterior cingulate cortex and ventromedial prefrontal cortex in autism: Evidence from coordinate-based imaging meta-analysis. Prog Neuropsychopharmacol Biol Psychiatry.

[56] Le TM, Potvin S, Zhornitsky S, Li C-SR (2021). Distinct patterns of prefrontal cortical disengagement during inhibitory control in addiction: a meta-analysis based on population characteristics. Neurosci Biobehav Rev.

[57] Lee JM, Kyeong S, Kim E, Cheon K-A (2016). Abnormalities of inter- and intra-hemispheric functional connectivity in autism spectrum disorders: a study using the autism brain imaging data exchange database. Front Neurosci.

[58] Leech R, Sharp DJ (2013). The role of the posterior cingulate cortex in cognition and disease. Brain.

[59] Li G, Rossbach K, Jiang W, Du Y (2018). Resting-state brain activity in chinese boys with low functioning autism spectrum disorder. Ann Gen Psychiatry.

[60] Li W, Pozzo-Miller L (2020). Dysfunction of the corticostriatal pathway in autism spectrum disorders. J Neurosci Res.

[61] Lian F, Northoff G (2021). The lost neural hierarchy of the autistic self—locked-out of the mental self and its default-mode network. Brain Sci.

[62] Liloia D, Cauda F, Uddin LQ, Manuello J, Mancuso L, Keller R, Nani A, Costa T (2022) Revealing the selectivity of neuroanatomical alteration in autism spectrum disorder via reverse inference. Biological psychiatry Cognitive neuroscience and neuroimaging 10.1016/j.bpsc.2022.01.007 35131520

[63] Liloia D, Mancuso L, Uddin LQ, Costa T, Nani A, Keller R, Manuello J, Duca S, Cauda F (2021). Gray matter abnormalities follow non-random patterns of co-alteration in autism: Meta-connectomic evidence. NeuroImage Clin.

[64] Liu J, Li Y, Yang X, Xu H, Ren J, Zhou P (2021). Regional spontaneous neural activity alterations in type 2 diabetes mellitus: a meta-analysis of resting-state functional mri studies. Front Aging Neurosci.

[65] Liu J, Zhang B, Wilson G, Kong J, Asdni T, Weiner MW, Aisen P, Weiner M, Aisen P, Petersen R, Jack CR, Jagust W, Trojanowki JQ, Toga AW, Beckett L, Green RC, Saykin AJ, Morris J, Shaw LM, Khachaturian Z, Sorensen G, Carrillo M, Kuller L, Raichle M, Paul S, Davies P, Fillit H, Hefti F, Holtzman D, Mesulam MM, Potter W, Snyder P, Schwartz A, Green RC, Montine T, Petersen R, Aisen P, Thomas RG, Donohue M, Walter S, Gessert D, Sather T, Jiminez G, Balasubramanian AB, Mason J, Sim I, Beckett L, Harvey D, Donohue M, Jack CR, Bernstein M, Fox N, Thompson P, Schuff N, DeCArli C, Borowski B, Gunter J, Senjem M, Vemuri P, Jones D, Kantarci K, Ward C, Jagust W, Koeppe RA, Foster N, Reiman EM, Chen K, Mathis C, Landau S, Morris JC, Cairns NJ, Franklin E, Taylor-Reinwald L, Shaw LM, Trojanowki JQ, Lee V, Korecka M, Figurski M, Toga AW, Crawford K, Neu S, Saykin AJ, Foroud TM, Potkin S, Shen L, Faber K, Kim S, Nho K, Weiner MW, Thal L, Khachaturian Z, Thal L, Buckholtz N, Weiner MW, Snyder PJ, Potter W, Paul S, Albert M, Frank R, Khachaturian Z (2019). New perspective for non-invasive brain stimulation site selection in mild cognitive impairment: Based on meta- and functional connectivity analyses. Frontiers in Aging Neuroscience.

[66] Loomes R, Hull L, Mandy WPL (2017). What is the male-to-female ratio in autism spectrum disorder? A systematic review and meta-analysis. J Am Acad Child Adolesc Psychiatry.

[67] Lord C, Brugha TS, Charman T, Cusack J, Dumas G, Frazier T, Jones EJH, Jones RM, Pickles A, State MW, Taylor JL, Veenstra-VanderWeele J (2020). Autism spectrum disorder. Nat Rev Dis Primers.

[68] Lord C, Charman T, Havdahl A, Carbone P, Anagnostou E, Boyd B, Carr T, de Vries PJ, Dissanayake C, Divan G, Freitag CM, Gotelli MM, Kasari C, Knapp M, Mundy P, Plank A, Scahill L, Servili C, Shattuck P, Simonoff E, Singer AT, Slonims V, Wang PP, Ysrraelit MC, Jellett R, Pickles A, Cusack J, Howlin P, Szatmari P, Holbrook A, Toolan C, McCauley JB (2022). The lancet commission on the future of care and clinical research in autism. Lancet.

[69] Lord C, Risi S, Lambrecht L, Cook EH, Leventhal BL, DiLavore PC, Pickles A, Rutter M (2000). The autism diagnostic observation schedule-generic: a standard measure of social and communication deficits associated with the spectrum of autism. J Autism Dev Disord.

[70] Lord C, Rutter M, Le Couteur A (1994). Autism diagnostic interview-revised: A revised version of a diagnostic interview for caregivers of individuals with possible pervasive developmental disorders. J Autism Dev Disord.

[71] Lynch CJ, Uddin LQ, Supekar K, Khouzam A, Phillips J, Menon V (2013). Default mode network in childhood autism: posteromedial cortex heterogeneity and relationship with social deficits. Biol Psychiat.

[72] Manuello J, Costa T, Cauda F, Liloia D (2022) Six actions to improve detection of critical features for neuroimaging coordinate-based meta-analysis preparation. Neuroscience & Biobehavioral Reviews:104659 10.1016/j.neubiorev.2022.104659 35405181

[73] Maximo J, Keown C, Nair A, Müller R-A (2013). Approaches to local connectivity in autism using resting state functional connectivity mri. Front Human Neurosci.

[74] Minshew NJ, Williams DL (2007). The new neurobiology of autism: cortex, connectivity, and neuronal organization. Arch Neurol.

[75] Monk CS, Peltier SJ, Wiggins JL, Weng SJ, Carrasco M, Risi S, Lord C (2009). Abnormalities of intrinsic functional connectivity in autism spectrum disorders. Neuroimage.

[76] Moreau CA, Harvey A, Kumar K, Huguet G, Urchs S, Douard EA, Schultz LM, Sharmarke H, Jizi K, Martin C-O, Younis N, Tamer P, Rolland T, Martineau J-L, Orban P, Silva AI, Hall J, van den Bree MBM, Owen MJ, Linden DEJ, Labbe A, Lippé S, Bearden CE, Almasy L, Glahn DC, Thompson PM, Bourgeron T, Bellec P, Jacquemont S (2022). Genetic heterogeneity shapes brain connectivity in psychiatry. Biol Psychiatry.

[77] Mueller S, Keeser D, Samson AC, Kirsch V, Blautzik J, Grothe M, Erat O, Hegenloh M, Coates U, Reiser MF, Hennig-Fast K, Meindl T (2013). Convergent findings of altered functional and structural brain connectivity in individuals with high functioning autism: a multimodal mri study. PLoS ONE.

[78] Muller VI, Cieslik EC, Laird AR, Fox PT, Radua J, Mataix-Cols D, Tench CR, Yarkoni T, Nichols TE, Turkeltaub PE, Wager TD, Eickhoff SB (2018). Ten simple rules for neuroimaging meta-analysis. Neurosci Biobehav Rev.

[79] Murias M, Webb SJ, Greenson J, Dawson G (2007). Resting state cortical connectivity reflected in eeg coherence in individuals with autism. Biol Psychiat.

[80] Nair S, Jao Keehn RJ, Berkebile MM, Maximo JO, Witkowska N, Müller RA (2018). Local resting state functional connectivity in autism: site and cohort variability and the effect of eye status. Brain Imaging Behav.

[81] Nijhuis EH, van Cappellen van Walsum AM, Norris DG,  (2013). Topographic hub maps of the human structural neocortical network. PLoS ONE.

[82] Nomi JS, Uddin LQ (2015). Developmental changes in large-scale network connectivity in autism. Neuroimage Clin.

[83] Noriuchi M, Kikuchi Y, Yoshiura T, Kira R, Shigeto H, Hara T, Tobimatsu S, Kamio Y (2010). Altered white matter fractional anisotropy and social impairment in children with autism spectrum disorder. Brain Res.

[84] Nunes AS, Vakorin VA, Kozhemiako N, Peatfield N, Ribary U, Doesburg SM (2020). Atypical age-related changes in cortical thickness in autism spectrum disorder. Sci Rep.

[85] O'Reilly C, Lewis JD, Elsabbagh M (2017). Is functional brain connectivity atypical in autism? A systematic review of eeg and meg studies. PLoS ONE.

[86] Oblak AL, Rosene DL, Kemper TL, Bauman ML, Blatt GJ (2011). Altered posterior cingulate cortical cyctoarchitecture, but normal density of neurons and interneurons in the posterior cingulate cortex and fusiform gyrus in autism. Autism Res.

[87] Paakki JJ, Rahko J, Long X, Moilanen I, Tervonen O, Nikkinen J, Starck T, Remes J, Hurtig T, Haapsamo H, Jussila K, Kuusikko-Gauffin S, Mattila ML, Zang Y, Kiviniemi V (2010). Alterations in regional homogeneity of resting-state brain activity in autism spectrum disorders. Brain Res.

[88] Padmanabhan A, Lynch CJ, Schaer M, Menon V (2017). The default mode network in autism. Biol Psychiatry Cognit Neurosci Neuroimaging.

[89] Pagani M, Bertero A, Liska A, Galbusera A, Sabbioni M, Barsotti N, Colenbier N, Marinazzo D, Scattoni ML, Pasqualetti M, Gozzi A (2019). Deletion of autism risk gene shank3 disrupts prefrontal connectivity. J Neurosci.

[90] Page MJ, McKenzie JE, Bossuyt PM, Boutron I, Hoffmann TC, Mulrow CD, Shamseer L, Tetzlaff JM, Akl EA, Brennan SE, Chou R, Glanville J, Grimshaw JM, Hróbjartsson A, Lalu MM, Li T, Loder EW, Mayo-Wilson E, McDonald S, McGuinness LA, Stewart LA, Thomas J, Tricco AC, Welch VA, Whiting P, Moher D (2021). The prisma 2020 statement: an updated guideline for reporting systematic reviews. BMJ.

[91] Pan P, Zhan H, Xia M, Zhang Y, Guan D, Xu Y (2017). Aberrant regional homogeneity in parkinson's disease: a voxel-wise meta-analysis of resting-state functional magnetic resonance imaging studies. Neurosci Biobehav Rev.

[92] Petinou K, Minaidou D (2017). Neurobiological bases of autism spectrum disorders and implications for early intervention: a brief overview. Folia Phoniatr Logop.

[93] Poldrack RA, Kittur A, Kalar D, Miller E, Seppa C, Gil Y, Parker DS, Sabb FW, Bilder RM (2011). The cognitive atlas: toward a knowledge foundation for cognitive neuroscience. Front Neuroinform.

[94] Radua J, Mataix-Cols D, Phillips ML, El-Hage W, Kronhaus DM, Cardoner N, Surguladze S (2012). A new meta-analytic method for neuroimaging studies that combines reported peak coordinates and statistical parametric maps. Eur Psychiatry.

[95] Radua J, Rubia K, Canales-Rodríguez EJ, Pomarol-Clotet E, Fusar-Poli P, Mataix-Cols D (2014). Anisotropic kernels for coordinate-based meta-analyses of neuroimaging studies. Front Psych.

[96] Riglin L, Wootton RE, Thapar AK, Livingston LA, Langley K, Collishaw S, Tagg J, Smith GD, Stergiakouli E, Tilling K, Thapar A (2021). Variable emergence of autism spectrum disorder symptoms from childhood to early adulthood. Am J Psychiatry.

[97] Rippon G, Brock J, Brown C, Boucher J (2007). Disordered connectivity in the autistic brain: Challenges for the "new psychophysiology". Int J Psychophysiol.

[98] Sarkheil P, Goebel R, Schneider F, Mathiak K (2013). Emotion unfolded by motion: a role for parietal lobe in decoding dynamic facial expressions. Soc Cogn Affect Neurosci.

[99] Sauer AK, Stanton JE, Hans S, Grabrucker AM, Grabrucker AM (2021). Autism spectrum disorders: Etiology and pathology. Autism spectrum disorders.

[100] Schacter DL, Addis DR (2007). The cognitive neuroscience of constructive memory: remembering the past and imagining the future. Philos Trans R Soc Lond B Biol Sci.

[101] Schulte-Rüther M, Greimel E, Markowitsch HJ, Kamp-Becker I, Remschmidt H, Fink GR, Piefke M (2011). Dysfunctions in brain networks supporting empathy: an fmri study in adults with autism spectrum disorders. Soc Neurosci.

[102] Seminowicz DA, Davis KD (2006). Interactions of pain intensity and cognitive load: the brain stays on task. Cereb Cortex.

[103] Shukla DK, Keehn B, Müller RA (2010). Regional homogeneity of fmri time series in autism spectrum disorders. Neurosci Lett.

[104] Smith SM, Nichols TE (2009). Threshold-free cluster enhancement: Addressing problems of smoothing, threshold dependence and localisation in cluster inference. Neuroimage.

[105] Song Y, Nie T, Shi W, Zhao X, Yang Y (2019). Empathy impairment in individuals with autism spectrum conditions from a multidimensional perspective: a meta-analysis. Front Psychol.

[106] Sporns O, Honey CJ, Kötter R (2007). Identification and classification of hubs in brain networks. PLoS ONE.

[107] Su T, Gong J, Tang G, Qiu S, Chen P, Chen G, Wang J, Huang L, Wang Y (2021). Structural and functional brain alterations in anorexia nervosa: a multimodal meta-analysis of neuroimaging studies. Hum Brain Mapp.

[108] Supekar K, Uddin LQ, Khouzam A, Phillips J, Gaillard WD, Kenworthy LE, Yerys BE, Vaidya CJ, Menon V (2013). Brain hyperconnectivity in children with autism and its links to social deficits. Cell Rep.

[109] Tahmasian M, Sepehry AA, Samea F, Khodadadifar T, Soltaninejad Z, Javaheripour N, Khazaie H, Zarei M, Eickhoff SB, Eickhoff CR (2019). Practical recommendations to conduct a neuroimaging meta-analysis for neuropsychiatric disorders. Hum Brain Mapp.

[110] Tomasi D, Wang GJ, Volkow ND (2013). Energetic cost of brain functional connectivity. Proc Natl Acad Sci U S A.

[111] Tudor ME, Walsh CE, Mulder EC, Lerner MD (2015). Pain as a predictor of sleep problems in youth with autism spectrum disorders. Autism.

[112] Tyszka JM, Kennedy DP, Paul LK, Adolphs R (2014). Largely typical patterns of resting-state functional connectivity in high-functioning adults with autism. Cereb cortex.

[113] Uddin L, Supekar K, Menon V (2013). Reconceptualizing functional brain connectivity in autism from a developmental perspective. Front Human Neurosci.

[114] Uddin LQ (2011). The self in autism: an emerging view from neuroimaging. Neurocase.

[115] Uddin LQ, Kelly AM, Biswal BB, Castellanos FX, Milham MP (2009). Functional connectivity of default mode network components: correlation, anticorrelation, and causality. Hum Brain Mapp.

[116] Uddin LQ, Menon V, Young CB, Ryali S, Chen T, Khouzam A, Minshew NJ, Hardan AY (2011). Multivariate searchlight classification of structural magnetic resonance imaging in children and adolescents with autism. Biol Psychiat.

[117] Uddin LQ, Supekar K, Lynch CJ, Khouzam A, Phillips J, Feinstein C, Ryali S, Menon V (2013). Salience network-based classification and prediction of symptom severity in children with autism. JAMA Psychiat.

[118] Valk SL, Di Martino A, Milham MP, Bernhardt BC (2015). Multicenter mapping of structural network alterations in autism. Hum Brain Mapp.

[119] van den Heuvel MP, Sporns O (2019). A cross-disorder connectome landscape of brain dysconnectivity. Nat Rev Neurosci.

[120] Vissers ME, Cohen MX, Geurts HM (2012). Brain connectivity and high functioning autism: a promising path of research that needs refined models, methodological convergence, and stronger behavioral links. Neurosci Biobehav Rev.

[121] von dem Hagen EA, Stoyanova RS, Baron-Cohen S, Calder AJ (2013). Reduced functional connectivity within and between 'social' resting state networks in autism spectrum conditions. Soc Cogn Affect Neurosci.

[122] Walker L, Gozzi M, Lenroot R, Thurm A, Behseta B, Swedo S, Pierpaoli C (2012). Diffusion tensor imaging in young children with autism: biological effects and potential confounds. Biol Psychiat.

[123] Wang J, Barstein J, Ethridge LE, Mosconi MW, Takarae Y, Sweeney JA (2013). Resting state eeg abnormalities in autism spectrum disorders. J Neurodev Disord.

[124] Wang W, Liu J, Shi S, Liu T, Ma L, Ma X, Tian J, Gong Q, Wang M (2018). Altered resting-state functional activity in patients with autism spectrum disorder: A quantitative meta-analysis. Front Neurol.

[125] Wantzen P, Clochon P, Doidy F, Wallois F, Mahmoudzadeh M, Desaunay P, Christian M, Guilé JM, Guénolé F, Eustache F, Baleyte JM, Guillery-Girard B (2022). Eeg resting-state functional connectivity: evidence for an imbalance of external/internal information integration in autism. J Neurodev Disord.

[126] Wass S (2011). Distortions and disconnections: disrupted brain connectivity in autism. Brain Cogn.

[127] Weng S-J, Wiggins JL, Peltier SJ, Carrasco M, Risi S, Lord C, Monk CS (2010). Alterations of resting state functional connectivity in the default network in adolescents with autism spectrum disorders. Brain Res.

[128] White SJ, Frith U, Rellecke J, Al-Noor Z, Gilbert SJ (2014). Autistic adolescents show atypical activation of the brain′s mentalizing system even without a prior history of mentalizing problems. Neuropsychol.

[129] Whitney DG, Shapiro DN (2019). National prevalence of pain among children and adolescents with autism spectrum disorders. JAMA Pediatr.

[130] Winkler AM, Ridgway GR, Webster MA, Smith SM, Nichols TE (2014). Permutation inference for the general linear model. Neuroimage.

[131] World Health Organization (1992). The icd-10 classification of mental and behavioural disorders : Clinical descriptions and diagnostic guidelines.

[132] Xu Y, Zhuo C, Qin W, Zhu J, Yu C (2015). Altered spontaneous brain activity in schizophrenia: a meta-analysis and a large-sample study. Biomed Res Int.

[133] Yarkoni T, Poldrack RA, Nichols TE, Van Essen DC, Wager TD (2011). Large-scale automated synthesis of human functional neuroimaging data. Nat Methods.

[134] Yasuda Y, Hashimoto R, Nakae A, Kang H, Ohi K, Yamamori H, Fujimoto M, Hagihira S, Takeda M (2016). Sensory cognitive abnormalities of pain in autism spectrum disorder: a case–control study. Ann Gen Psychiatry.

[135] Yeo BTT, Krienen FM, Sepulcre J, Sabuncu MR, Lashkari D, Hollinshead M, Roffman JL, Smoller JW, Zöllei L, Polimeni JR, Fischl B, Liu H, Buckner RL (2011). The organization of the human cerebral cortex estimated by intrinsic functional connectivity. J Neurophysiol.

[136] You X, Norr M, Murphy E, Kuschner E, Bal E, Gaillard W, Kenworthy L, Vaidya C (2013). Atypical modulation of distant functional connectivity by cognitive state in children with autism spectrum disorders. Front Human Neurosci.

[137] Zang Y, Jiang T, Lu Y, He Y, Tian L (2004). Regional homogeneity approach to fmri data analysis. Neuroimage.

[138] Zhang B, Liu J, Bao T, Wilson G, Park J, Zhao B, Kong J (2020). Locations for noninvasive brain stimulation in treating depressive disorders: a combination of meta-analysis and resting-state functional connectivity analysis. Aust N Z J Psychiatry.

[139] Zhang X, Li L, Huang G, Zhang L, Liang Z, Shi L, Zhang Z (2021). A multisensory fmri investigation of nociceptive-preferential cortical regions and responses. Front Neurosci.

[140] Zuo XN, Ehmke R, Mennes M, Imperati D, Castellanos FX, Sporns O, Milham MP (2012). Network centrality in the human functional connectome. Cereb cortex.

[141] Zuo XN, Xu T, Jiang L, Yang Z, Cao XY, He Y, Zang YF, Castellanos FX, Milham MP (2013). Toward reliable characterization of functional homogeneity in the human brain: preprocessing, scan duration, imaging resolution and computational space. Neuroimage.

