# Supplementary material for: Atypical local brain connectivity in pediatric autism spectrum disorder? A coordinate-based meta-analysis of regional homogeneity studies
Source: Eur Arch Psychiatry Clin Neurosci. 2023 Jan 4;274(1):3–18. doi: 10.1007/s00406-022-01541-2 (PMC10787009; doi:10.1007/s00406-022-01541-2)
Supplement: Supplementary file 1 — Supplementary file1 (DOCX 668 KB) [file 406_2022_1541_MOESM1_ESM.docx]

**Atypical Local Brain Connectivity in Pediatric Autism Spectrum Disorder?**

**A Coordinate-Based Meta-Analysis of Regional Homogeneity Studies**

Supplementary Material

*Table S1.* PRISMA 2020 Checklist…p.2

*Table S2.* Full search strategies for data selection…p.5

*Table S3.* Medical comorbidities in autism spectrum disorder groups for each included ReHo experiment… p.7

*Table S4.* Rationale of inclusion and exclusion for experiments with subjects from ABIDE database…p.8

*Table S5.* Regional homogeneity changes in pediatric individuals with autism spectrum disorder compared with typically developmental controls at P = 0.005 uncorrected…p.9

*Figure S1.* Brain clusters of regional homogeneity changes in pediatric individuals with autism spectrum disorder compared with typically developmental controls at P = 0.005 uncorrected…p.10

*Table S6.* Concept definition for each individuated psychological term as provided by the Cognitive Atlas ontology (<https://www.cognitiveatlas.org/>)…p.11

*Table S1.* PRISMA 2020 Checklist.

| **Section and Topic** | **Item #** | **Checklist item** | **Section where item is reported** |
| --- | --- | --- | --- |
| **TITLE** | | |  |
| Title | 1 | Identify the report as a systematic review or meta-analysis. | TITLE |
| **ABSTRACT** | | |  |
| Abstract | 2 | See the PRISMA 2020 for Abstracts checklist. | ABSTRACT |
| **INTRODUCTION** | | |  |
| Rationale | 3 | Describe the rationale for the review in the context of existing knowledge. | INTRODUCTION |
| Objectives | 4 | Provide an explicit statement of the objective(s) or question(s) the review addresses. |  |
| **METHODS** | | |  |
| Eligibility criteria | 5 | Specify the inclusion and exclusion criteria for the review and how studies were grouped for the syntheses. | SEARCH STRATEGY AND DATA |
| Information sources | 6 | Specify all databases, registers, websites, organizations, reference lists and other sources searched or consulted to identify studies. Specify the date when each source was last searched or consulted. |  |
| Search strategy | 7 | Present the full search strategies for all databases, registers and websites, including any filters and limits used. | TABLE S2 |
| Selection process | 8 | Specify the methods used to decide whether a study met the inclusion criteria of the review, including how many reviewers screened each record and each report retrieved, whether they worked independently, and if applicable, details of automation tools used in the process. | SEARCH STRATEGY AND DATA SELECTION |
| Data collection process | 9 | Specify the methods used to collect data from reports, including how many reviewers collected data from each report, whether they worked independently, any processes for obtaining or confirming data from study investigators, and if applicable, details of automation tools used in the process. |  |
| Data items | 10a | List and define all outcomes for which data were sought. Specify whether all results that were compatible with each outcome domain in each study were sought (e.g. for all measures, time points, analyses), and if not, the methods used to decide which results to collect. |  |
|  | 10b | List and define all other variables for which data were sought (e.g. participant and intervention characteristics, funding sources). Describe any assumptions made about any missing or unclear information. |  |
| Study risk of bias assessment | 11 | Specify the methods used to assess risk of bias in the included studies, including details of the tool(s) used, how many reviewers assessed each study and whether they worked independently, and if applicable, details of automation tools used in the process. | HETEROGENEITY AND PUBLICATION BIAS EVALUATION |
| Effect measures | 12 | Specify for each outcome the effect measure(s) (e.g. risk ratio, mean difference) used in the synthesis or presentation of results. |  |
| Synthesis methods | 13a | Describe the processes used to decide which studies were eligible for each synthesis (e.g. tabulating the study intervention characteristics and comparing against the planned groups for each synthesis (item #5)). | SEARCH STRATEGY AND DATA SELECTION |
|  | 13b | Describe any methods required to prepare the data for presentation or synthesis, such as handling of missing summary statistics, or data conversions. | DATA EXTRACTION |
|  | 13c | Describe any methods used to tabulate or visually display results of individual studies and syntheses. | COORDINATE-BASED META-ANALYSIS |
|  | 13d | Describe any methods used to synthesize results and provide a rationale for the choice(s). If meta-analysis was performed, describe the model(s), method(s) to identify the presence and extent of statistical heterogeneity, and software package(s) used. | STATISTICAL METHODS SECTIONS |
|  | 13e | Describe any methods used to explore possible causes of heterogeneity among study results (e.g. subgroup analysis, meta-regression). | HETEROGENEITY AND PUBLICATION BIAS EVALUATION |
|  | 13f | Describe any sensitivity analyses conducted to assess robustness of the synthesized results. |  |
| Reporting bias assessment | 14 | Describe any methods used to assess risk of bias due to missing results in a synthesis (arising from reporting biases). |  |
| Certainty assessment | 15 | Describe any methods used to assess certainty (or confidence) in the body of evidence for an outcome. | META-REGRESSION ANALYSES |
| **RESULTS** | | |  |
| Study selection | 16a | Describe the results of the search and selection process, from the number of records identified in the search to the number of studies included in the review, ideally using a flow diagram. | RESULTS |
|  | 16b | Cite studies that might appear to meet the inclusion criteria, but which were excluded, and explain why they were excluded. |  |
| Study characteristics | 17 | Cite each included study and present its characteristics. |  |
| Risk of bias in studies | 18 | Present assessments of risk of bias for each included study. | ANALYSIS OF HETEROGENEITY AND PUBLICATION BIAS |
| Results of individual studies | 19 | For all outcomes, present, for each study: (a) summary statistics for each group (where appropriate) and (b) an effect estimate and its precision (e.g. confidence/credible interval), ideally using structured tables or plots. | RESULTS SECTIONS |
| Results of syntheses | 20a | For each synthesis, briefly summarize the characteristics and risk of bias among contributing studies. | RESULTS  ANALYSIS OF HETEROGENEITY AND PUBLICATION BIAS |
|  | 20b | Present results of all statistical syntheses conducted. If meta-analysis was done, present for each the summary estimate and its precision (e.g. confidence/credible interval) and measures of statistical heterogeneity. If comparing groups, describe the direction of the effect. |  |
|  | 20c | Present results of all investigations of possible causes of heterogeneity among study results. |  |
|  | 20d | Present results of all sensitivity analyses conducted to assess the robustness of the synthesized results. |  |
| Reporting biases | 21 | Present assessments of risk of bias due to missing results (arising from reporting biases) for each synthesis assessed. |  |
| Certainty of evidence | 22 | Present assessments of certainty (or confidence) in the body of evidence for each outcome assessed. |  |
| **DISCUSSION** | | |  |
| Discussion | 23a | Provide a general interpretation of the results in the context of other evidence. | DISCUSSION |
|  | 23b | Discuss any limitations of the evidence included in the review. | LIMITATIONS AND FUTURE CHALLENGES |
|  | 23c | Discuss any limitations of the review processes used. | DISCUSSION |
|  | 23d | Discuss implications of the results for practice, policy, and future research. | DISCUSSION  LIMITATIONS AND FUTURE CHALLENGES |
| **OTHER INFORMATION** | | |  |
| Registration and protocol | 24a | Provide registration information for the review, including register name and registration number, or state that the review was not registered. | N/A |
|  | 24b | Indicate where the review protocol can be accessed, or state that a protocol was not prepared. |  |
|  | 24c | Describe and explain any amendments to information provided at registration or in the protocol. |  |
| Support | 25 | Describe sources of financial or non-financial support for the review, and the role of the funders or sponsors in the review. | ACKNOWLEDGMENTS |
| Competing interests | 26 | Declare any competing interests of review authors. |  |
| Availability of data, code and other materials | 27 | Report which of the following are publicly available and where they can be found: template data collection forms; data extracted from included studies; data used for all analyses; analytic code; any other materials used in the review. |  |

*Table S2.* Full search strategies for data selection.

| *A.*  *Systematic literature search on a*  *bibliographic database* | **Databases**: PubMed MEDLINE (<https://www.ncbi.nlm.nih.gov/pubmed/>)  **PubMed Advanced Search Builder**:  (((((autism[Title/Abstract]) OR (autism spectrum disorder[Title/Abstract])) OR (ASD[Title/Abstract])) AND (regional homogeneity[Title/Abstract])) OR (ReHo[Title/Abstract])) OR (local connectivity[Title/Abstract]) |
| --- | --- |
| *B.*  *Reference list check of included articles* | *Articles screened:*  *1.* Dajani, D. R., & Uddin, L. Q. (2016). Local brain connectivity across development in autism spectrum disorder: A cross-sectional investigation. *Autism Res, 9*(1), 43-54. doi:10.1002/aur.1494  **2.** Floris, D. L., Filho, J. O. A., Lai, M. C., Giavasis, S., Oldehinkel, M., Mennes, M., . . . Di Martino, A. (2021). Towards robust and replicable sex differences in the intrinsic brain function of autism. *Mol Autism, 12*(1), 19. doi:10.1186/s13229-021-00415-z  **3.** Jao Keehn, R. J., Nair, S., Pueschel, E. B., Linke, A. C., Fishman, I., & Müller, R. A. (2019). Atypical Local and Distal Patterns of Occipito-frontal Functional Connectivity are Related to Symptom Severity in Autism. *Cereb Cortex, 29*(8), 3319-3330. doi:10.1093/cercor/bhy201  **4.** Lan, Z., Xu, S., Wu, Y., Xia, L., Hua, K., Li, M., . . . Wang, T. (2021). Alterations of Regional Homogeneity in Preschool Boys With Autism Spectrum Disorders. *Front Neurosci, 15*, 644543. doi:10.3389/fnins.2021.644543  **5.** Li, G., Rossbach, K., Jiang, W., & Du, Y. (2018). Resting-state brain activity in Chinese boys with low functioning autism spectrum disorder. *Ann Gen Psychiatry, 17*, 47. doi:10.1186/s12991-018-0217-z  **6.** Maximo, J., Keown, C., Nair, A., & Müller, R.-A. (2013). Approaches to local connectivity in autism using resting state functional connectivity MRI. *Frontiers in Human Neuroscience, 7*(605). doi:10.3389/fnhum.2013.00605  **7.** Nair, S., Jao Keehn, R. J., Berkebile, M. M., Maximo, J. O., Witkowska, N., & Müller, R. A. (2018). Local resting state functional connectivity in autism: site and cohort variability and the effect of eye status. *Brain Imaging Behav, 12*(1), 168-179. doi:10.1007/s11682-017-9678-y  **8.** Paakki, J. J., Rahko, J., Long, X., Moilanen, I., Tervonen, O., Nikkinen, J., . . . Kiviniemi, V. (2010). Alterations in regional homogeneity of resting-state brain activity in autism spectrum disorders. *Brain Res, 1321*, 169-179. doi:10.1016/j.brainres.2009.12.081 |
| *C.*  *Reference list check of relevant reviews* | *Reviews screened:*  *1.* Hull, J. V., Dokovna, L. B., Jacokes, Z. J., Torgerson, C. M., Irimia, A., & Van Horn, J. D. (2017). Resting-State Functional Connectivity in Autism Spectrum Disorders: A Review. *Front Psychiatry, 7*(205). doi:10.3389/fpsyt.2016.00205  **2.** Lian, F., & Northoff, G. (2021). The Lost Neural Hierarchy of the Autistic Self—Locked-Out of the Mental Self and Its Default-Mode Network. *Brain Sciences, 11*(5), 574. doi: 10.3390/brainsci11050574 |

*Table S3.* Medical comorbidities in groups with autism spectrum disorder for each included ReHo experiment.

| **ReHo experiments** | **Co-occurrence of** | | |
| --- | --- | --- | --- |
|  | **Neurological**  **conditions** | **Psychiatric**  **disorders** | **Genetic**  **disorders** |
| Dajani 2016 (children) | No | No | Not specified |
| Dajani 2016 (adolescent) | No | No | Not specified |
| Floris 2021 | No | No | No |
| Jao Keehn 2019 | No | Not specified | No |
| Lan 2021 | No | No | No |
| Li 2018 | Not specified | No | Not specified |
| Maximo 2013 | No | Not specified | No |
| Nair 2018 (SDSU) | No | Not specified | No |
| Nair 2018 (ABIDE-EO) | No | No | No* |
| Nair 2018 (ABIDE-EC) | No | No | No** |
| Paakki 2010 | No | No | Not specified |

*Not specified, information not reported in the original article.*

** Information provided only for 26 of 59 subjects with ASD (i.e. UCLA1, UCLA2, USM data sets). ** Information provided only for 19 of 30 subjects with ASD (i.e. PITT, Stanford, Trinity data sets).*

*Table S4.* Rationale of inclusion and exclusion for experiments with subjects from ABIDE database.

| Article | ReHo Experiment | ABIDE Database | ABIDE  Sites | Selection  Criteria |
| --- | --- | --- | --- | --- |
| Dajani & Uddin, 2016 | Children group | ABIDE I | NYU (eyes open during the scan session) | Included: Yes  No subjects’ overlap |
|  | Adolescent group | ABIDE I | NYU (eyes open during the scan session) | Included: Yes  No subjects’ overlap |
|  | Adult group | ABIDE I | NYU (eyes open during the scan session) | Included: No  Subjects’ age > 18 years old |
| Nair et al., 2018 | ABIDE  Eyes open group | ABIDE I | KKI, OHSU, OLIN, UCLA1, UCLA2, UM1, UM2, USM, Yale (eyes open during the scan session) | Included: Yes  No subjects’ overlap |
|  | ABIDE  Eyes closed group | ABIDE I | Leuven2, NYU, PITT, Stanford, Trinity (eyes closed during the scan session) | Included: Yes  No subjects’ overlap |
|  | SDSU  In-house group | N/A | N/A | Included: Yes  No subjects’ overlap  No subjects from ABIDE database |
| Floris et al., 2021 | ABIDE group | ABIDE I  ABIDE II | KKI, Leuven2, NYU, OHSU, Pitt, SDSU, Stanford, UCLA1, UM1, Yale, GU1, KKI1, KKI2, NYU1, OHSU1, SDSU1, UCD1, UCLA1 | Included: No  Possible subjects’ overlap with previous published articles included in the current meta-analysis |
|  | EU-AIMS group | N/A | N/A | Included: No  Subjects’ age > 18 years old |
|  | GENDAAR group | N/A | N/A | Included: Yes  No subjects’ overlap  No subjects from ABIDE database |

*Table S4.* Regional homogeneity changes in pediatric individuals with autism spectrum disorder compared with typically developmental controls at P = 0.005 uncorrected.

| **Region** | **MNI coordinate** | | | **SDM** | ***P < 0.005*** | **Voxels** | **Cluster breakdown** |
| --- | --- | --- | --- | --- | --- | --- | --- |
|  | x | y | z | ***Z* score** | ***(Uncorrected)*** |  | **(Voxels)** |
| **ASD > TDCs** | | | | | | | |
| Left calcarine cortex  (BA 17) | 2 | -86 | 2 | 3.414 | 0.0003 | 65 | Left Cal BA 17 (36)  Right Cal BA 17 (18)  Bilateral LG BA 17 (11) |
| **ASD < TDCs**  Right paracentral lobule  (BA 4) | 6 | -32 | 60 | -4.708 | 0.000001 | 1692 | Right PCL BAs 4/5 (483)  Left MCC BA 23 (368)  Right MCC BA 23 (292)  Left PCL BAs 4/5 (149)  Left PCC BAs 23/30 (131)  Left Pcun BAs 5/23 (131)  Right SMA BAs 4/6 (63)  Right Pcun BAs 5/23 (35)  Right PoCG BA 4 (18)  Right PCC BA 23 (14)  Left SMA BAs 4/6 (8) |
| Right superior frontal gyrus  (BA 10) | 2 | 56 | -2 | -5.082 | 0.0000001 | 817 | Right SFG BAs 10/11 (455)  Left SFG BAs 10/11 (179)  Left ACC BAs 10/32 (109)  Right ACC BAs 10/32 (74) |
|  |  |  |  |  |  |  |  |
| Abbreviations: ASD, autism spectrum disorder; TDCs, typically developing controls; BA, Brodmann area; MNI, Montreal Neurological Institute; SDM, Seed-based d Mapping; Cal, calcarine cortex; LG, lingual gyrus; PCL, paracentral lobule; MCC, median cingulate cortex; PCC, posterior cingulate cortex; PCUN, precuneus; SMA, supplementary motor area; PoCG, posterior central gyrus; SFG, superior frontal gyrus; ACC, anterior cingulate cortex. | | | | | | | |

***
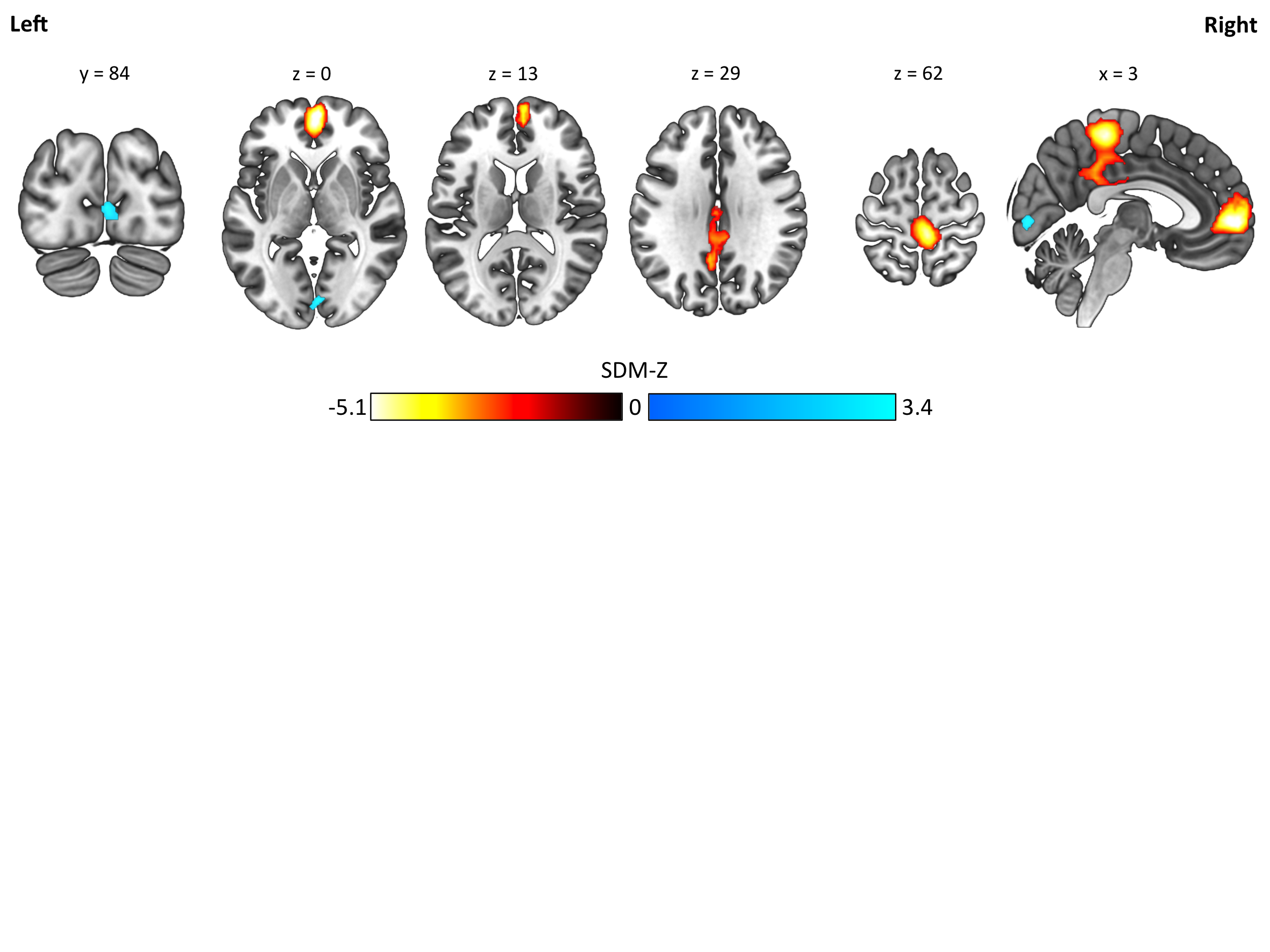
***

*Figure S1.* Brain clusters of regional homogeneity changes in pediatric individuals with autism spectrum disorder compared with typically developmental controls at P = 0.005 uncorrected.

*The PSI-SDM map is visualized as six axial slices (2-D cortical and subcortical view). Colors from red to yellow represent a local hypoconnectivity (regional homogeneity in autism spectrum disorder < typically developmental controls). Colors from dark to light blue represent a local hyperconnectivity (regional homogeneity in autism spectrum disorder > typically developmental controls). Brain templates are in neurological convention (i.e. Right is right, Left is left).*

*Table S5.* Concept definition for each individuated psychological term as provided by the Cognitive Atlas ontology (<https://www.cognitiveatlas.org/>).

| ***PSI-SDM Peak (coordinates)*** | ***Concept*** | ***Z-score*** | ***Cognitive Atlas Definition*** |
| --- | --- | --- | --- |
| Right PCL  (6, -32, 60) | Pain | 4.86 | An unpleasant sensory and emotional experience associated with actual or potential tissue damage. |
|  | Nociception | 4.12 | The processes of encoding and processing noxious stimuli. |
| Right SFG  (2, 56, -2) | Reward (valuation) | 6.1 | Processes by which the probability and benefits of a prospective outcome are computed and calibrated by reference to external information, social context (e.g., group input, counterfactual comparisons), and/or prior experience. This calibration is influenced by pre-existing biases, learning, memory, stimulus characteristics, and deprivation states. Reward valuation may involve the assignment of incentive salience to stimuli. |
|  | Autobiographical memory | 5.08 | A memory system consisting of episodes recollected from an individual’s life, based on a combination of episodic (personal experiences and specific objects, people and events experienced at particular time and place) and semantic (general knowledge and facts about the world) memory. |
| Left PCC  (-2, -50, 32) | Theory of Mind | 6.61 | The ability for a person to connect emotional states to themselves and others and understand that other people may have different beliefs, desires, or intentions from one's self. It is intimately connected with the development of a person's ability to analyze and interpret the intentions of others. |
|  | Mentalization | 5.95 | The ability to understand the mental state, of oneself or others, that underlies overt behavior. |
|  | Autobiographical memory | 4.09 | A memory system consisting of episodes recollected from an individual’s life, based on a combination of episodic (personal experiences and specific objects, people and events experienced at particular time and place) and semantic (general knowledge and facts about the world) memory. |
|  | Empathy | 4.05 | The act of understanding, being aware of, being sensitive to, and or experiencing the feelings, thoughts, and experience of another. |
